# Modern heart failure treatment is superior to conventional treatment across the left ventricular ejection spectrum: real-life data from the Swedish Heart Failure Registry 2013–2020

**DOI:** 10.1007/s00392-024-02498-z

**Published:** 2024-08-26

**Authors:** Patric Karlström, Aldina Pivodic, Ulf Dahlström, Michael Fu

**Affiliations:** 1 Department of Internal Medicine, Ryhov County Hospital, SE-551 85 Jönköping, Sweden; 2 Department of Health, Medicine and Caring Sciences, Linköping University, Linköping, Sweden; 3 APNC Sweden, Gothenburg, Sweden; 4 Department of Clinical Neuroscience, Institute of Neuroscience and Physiology, Sahlgrenska Academy, University of Gothenburg, Gothenburg, Sweden; 5 Department of Cardiology and Department of Health, Medicine and Caring Sciences, Linköping University, Linköping, Sweden; 6 Department of Molecular and Clinical Medicine, Institute of Medicine, Sahlgrenska Academy, Sahlgrenska University Hospital/Östra Hospital, University of Gothenburg, Gothenburg, Sweden

**Keywords:** Heart failure, Effectiveness, Real world, Sodium-glucose cotransporter 2 inhibitors (SGLT2i), Angiotensin receptor-neprilysin inhibitor (ARNI)

## Abstract

**Objectives:**

This study is aimed to compare the effectiveness of modern therapy including angiotensin receptor-neprilysin inhibitor (ARNI) and sodium-glucose cotransporter 2 inhibitors (SGLT2i) with conventional heart failure treatment in the real world.

**Background:**

Since ARNI and SGLT2i were introduced to treat heart failure (HF), its therapeutic regimen has modernized from previous treatment with beta-blocker (BB) and angiotensin-converting enzyme inhibitor (ACEi)/angiotensin II receptor blocker (ARB) with mineralocorticoid receptor antagonist (MRA) as added-on in HF with reduced ejection fraction (HFrEF). However, a comparison between conventional and modern treatment strategies with drugs in combination has not been performed.

**Methods:**

This observational study (2013–2020), using the Swedish HF Registry, involved 20,849 HF patients. Patients received either conventional (BB, ACEi/ARB, with/without MRA, *n* = 20,140) or modern (BB, ACEi/ARB, MRA, SGLT2i or BB, ARNI, MRA with/without SGLT2i, *n* = 709) treatment at the index visit. The endpoints were all-cause and cardiovascular (CV) mortality.

**Results:**

Modern HF therapy was associated with a significant 28% reduction in all-cause mortality (adjusted HR [aHR], 0.72 (0.54–0.96); *p* = 0.024) and a significant 62% reduction in CV mortality (aHR, 0.38 (0.21–0.68); *p* = 0.0013) compared to conventional HF treatment. Similar results emerged in a sensitivity analysis using propensity score matching. The interaction analyses did not reveal any trends for EF (< 40% and ≥ 40%), sex, age (< 70 and ≥ 70 years), eGFR (< 60 and ≥ 60 ml/min/1.73 m^2^), and etiology of HF subgroups.

**Conclusion:**

In this nationwide study, modern HF therapy was associated with significantly reduced all-cause and CV mortality, regardless of EF, sex, age, eGFR, and etiology of HF.

**Graphical abstract:**

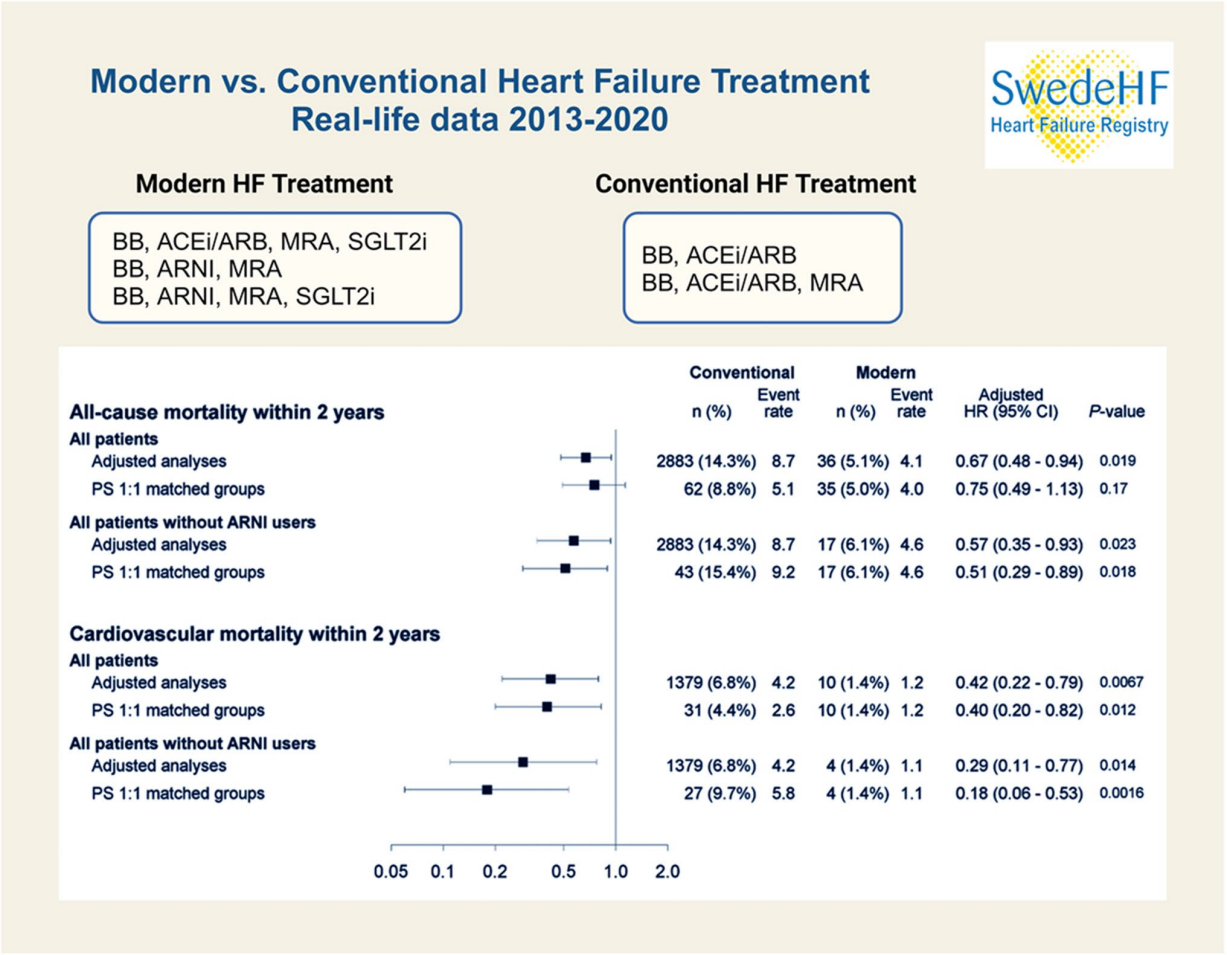

**Supplementary Information:**

The online version contains supplementary material available at 10.1007/s00392-024-02498-z.

## Introduction

Heart failure (HF) is a clinical syndrome characterized by high mortality and morbidity, as well as impaired quality of life (QoL) [1]. The global prevalence of HF is estimated to be about 64 million patients [2]. Previously, patients with HF with reduced ejection fraction (HFrEF) were recommended treatment with angiotensin-converting enzyme inhibitors (ACEi) or an angiotensin II receptor blocker (ARB) and beta-blocker (BB) combined with a mineralocorticoid receptor antagonist (MRA) [3].

In 2016, sacubitril/valsartan, an angiotensin receptor-neprilysin inhibitor (ARNI), was recommended as a new treatment option for HF in patients with HFrEF [4, 5]. The recommendations were based on the Prospective Comparison of ARNI with ACEI to Determine Impact on Global Mortality and Morbidity in Heart Failure (PARADIGM-HF) that showed ARNI to be superior to enalapril in patients with BB and MRA in reducing the risk of mortality and HF hospitalization [6].

After the Dapagliflozin and Prevention of Adverse Outcomes in Heart Failure (DAPA-HF) trial [7] and the Empagliflozin Outcome Trial in Patients with Chronic Heart Failure and a Reduced Ejection Fraction (EMPEROR-Reduced) [8] that demonstrated significantly reduced HF events or cardiovascular (CV) mortality, sodium-glucose cotransporter 2 inhibitors (SGLT2i) were introduced in the 2021 European Society of Cardiology guidelines for treatment of HFrEF patients [9].

The Dapagliflozin Evaluation to Improve the LIVEs of Patients With PReserved Ejection Fraction Heart Failure (DELIVER) trial [10] and the Empagliflozin Outcome Trial in Patients with Chronic Heart Failure with Preserved Ejection Fraction (EMPEROR-Preserved) [11] showed that both dapagliflozin and empagliflozin are effective in reducing HF events or CV mortality in HF with mildly reduced or preserved ejection fraction (EF) [10, 11]. A meta-analysis of five randomized controlled trials (RCTs) demonstrated that SGLT2i reduced the risk of composite CV mortality or hospitalization for HF, CV mortality, and all-cause mortality in a broad range of patients with HF, irrespective of EF [12].

Komajda et al. reported that the physicians’ adherence to guideline-recommended HF therapies is associated with improved outcome in patients with HFrEF [13]. In a cross-trial analysis, Vaduganathan et al. found that comprehensive HF treatment (ARNI, BB, MRA, SGLT2i) reduces CV mortality, HF hospitalization, and all-cause mortality compared to conventional HF medication (ACEi or ARB and BB) [14].

RCTs are the gold standard for the assessment of efficacy. Nonetheless, insights extracted from analyses conducted on real-world data play a vital role in supplementing our understanding. This study used patient-level data from the Swedish Heart Failure Registry (SwedeHF) linked to clinical outcomes derived from three other National Administrative Health Registries. The objective was to assess the real-world effectiveness of modern HF treatment in patients with HF compared to conventional HF treatment across a broad spectrum of EF.

Because each pharmacological agent was previously studied separately in a placebo-controlled randomized trial with different background therapies and study designs, the expected overall treatment benefit from combining several agents remains obscure. Moreover, a head-to-head comparison between conventional and modern therapeutic regimens is still lacking. Moreover, real-world data about strategy-based comparisons of the effectiveness between conventional and contemporary treatment regimens are unavailable.

## Methods

### Study material

The data for this retrospective observational study were drawn from the SwedeHF, which has been described elsewhere [15]. Since 2003, patients have been included in the SwedeHF. The only inclusion criterion was clinician-judged HF up to 2017 and defined since 2017 by the following International Classification of Diseases 10th revision (ICD-10) codes: I50.0, I50.1, I50.9, I42.0, I42.6, I42.7, I25.5, I11.0, I13.0, and I13.2. In 2020, approximately 31% of the prevalent HF population in Sweden was registered in the SwedeHF. About 80 variables are registered after an outpatient clinic visit or at discharge from the hospital and are entered into a database managed by the Uppsala Clinical Research Center, Uppsala, Sweden (Swedehf.se). To access diverse information (e.g., comorbidities, dates of death and their causes thereof) and medication dispensation from pharmacies, SwedeHF was linked to the Swedish National Patient Register, including both in- and outpatient visits, the Cause of Death Register, and the National Prescribed Drug Register, respectively, using the unique personal identification number assigned to all Swedish residents. The extracted comorbidities and reason for death were identified through the ICD-10 codes and medications through the Anatomical Therapeutic Chemical (ATC) classification as detailed in Supplementary Table 1.

The study adhered to the principles of the Declaration of Helsinki, and ethical approval was granted by the Swedish Ethical Review Authority. While individual patient consent was not required, all patients were informed about their inclusion in the SwedeHF and were given the choice to decline participation.

### Study patients

The study cohort comprised 20,849 patients of the 103,782 patients registered in the SwedeHF from 2003 to 2020. These 20,849 patients were undergoing treatment involving either conventional or modern HF therapies at the index visit. The following exclusion criteria were applied: patients with an index visit before September 3, 2013 (*n* = 53,019), as this is the first date of SGLT2i dispensation, those with HF duration exceeding 6 months (*n* = 25,048), individuals on other combinations of HF therapies than those targeted in the current study (*n* = 4765), and instances of data inconsistency (*n* = 101) (Supplementary Figure 1).

### Treatment definitions

Treatment regimens (modern vs. conventional) were defined based on guideline recommendations. The most related guideline recommendation for the study period 2003–2020 was the 2016 European Society of Cardiology (ESC) Heart Failure Guidelines, in which BB and ACEi/ARB were the first-line treatment of HFrEF, and MRA and ARNI were added-on therapy. As a result, MRA was prescribed only in approximately half of patients with HFrEF and even less with ARNI. The year 2021 is a turning point in guideline recommendation from sequential order to early or simultaneous initiation (BB, ACEI/ARB/ARNI, MRA, and SGLT2i) within 2 weeks. But our data were extracted from a time (2003–2020) when Guideline directed medical therapy (GDMT) has traditionally been implemented in the consecutive order by which they were added stepwise to the guidelines, e.g., starting ACEi/ARB + BB, followed by the addition of MRA. MRA has been added-on drug since 2008 through 2016 until 2021. In the meantime, ARNI has also been recommended in Sweden as added-on drug to replace ACEI/ARB, and SGLT2i was used mainly due to T2DM. Therefore, in order to reflect clinical practice in the real world, together with the availability of our database, the following definition was applied: conventional therapy was defined as having obligatory dual treatments with BB, ACEi/ARB as the backbone and with or without MRA as an option, whereas modern therapy must meet 3 criteria: (1) having obligatory at least triple treatments with BB, ACEi/ARB/ARNI, and MRA as the backbone and (2) containing either SGLT2i or ARNI or both. This approach involved categorizing patients into treatment groups based on medication information in the SwedeHF. Patients were assigned based on medication dispensation occurring within 3 months before or up to 6 months after the index registration in the SwedeHF. Medication data were retrieved from the SwedeHF and the Swedish Prescribed Drug Register, focusing on dispensation dates in relation to the index registration.

### Study endpoints

The study endpoints were risk reductions associated with all-cause and CV mortality, followed up from the index date until censoring or event of interest. Due to bias in the follow-up time between the HF therapy groups, the statistical analyses were also performed on the follow-up data within 2 years from the index date.

### Statistical analysis

Descriptively, continuous variables were presented as mean, standard deviation, median, and range, and categorical variables by frequency and percentage.

For the test between conventional and modern HF therapy for patients’ clinical characteristics and comorbidities, Fisher’s exact test was used for dichotomous variables, Mantel–Haenszel Chi-squared trend test for ordered categorical variables and the Mann–Whitney *U*-test for continuous variables.

Event rates were calculated as the number of events divided by the total number of follow-up years, expressed as 100 person-years. Lower and upper 95% confidence limits were estimated using exact Poisson limits.

Time-to-event analyses were performed using Cox proportional hazards models adjusted for clinically important risk factors and variables statistically significantly differing between the two therapies. Those variables were age, sex, body mass index (BMI) (< 18.5, 18.5–25. > 25–30, > 30–35, > 35 kg/m^2^), New York Heart Association (NYHA) functional classification, left ventricular EF (LVEF), N-terminal pro-B-type natriuretic peptide (NT-proBNP), estimated glomerular filtration rate (eGFR, < 60, ≥ 60 ml/min/1.73 m^2^), estimated by using the CKD-EPI algorithm [16], implantable cardioverter-defibrillator (ICD), cardiac resynchronization therapy (CRT), comorbidities (ischemic heart disease (IHD), any valve surgery or disease, hypertension, atrial fibrillation (AF), and diabetes mellitus (DM). Hazard Ratios (HRs) with 95% confidence intervals (CIs) were presented. The assumption of proportional hazards was tested using the interaction between the treatment group variable and the log (follow-up time) in the Cox regression models. The interaction analyses were performed for subgroups of LVEF < / ≥ 40%, any valve surgery or disease, IHD, sex, age < / ≥ 70 years, and eGFR < 60/ ≥ 60 ml/min by including an interaction term between the subgrouping variable and the HF therapy. Results were presented in forest plots.

For robustness, all analyses were repeated on propensity score 1:1 matched groups in two variants, including the variables age, sex, BMI, NYHA, LVEF, NT-proBNP, eGFR, IHD, valve surgery, hypertension, AF, DM, ICD, and CRT, with and without ARNI. The matching procedure, including ARNI, resulted in both treatment groups not having any patients on ARNI therapy, leaving only the effect of SGLT2i to be the target assessment. As a matching algorithm, a 1:1, nearest neighbor matching, with the optimal caliper width of 0.2 of the standard deviation of the logit of the propensity score, was used. To validate the performance of the matching procedure, the distribution of the propensity scores and patient characteristics and medical history were compared between the two HF therapies. Unadjusted cumulative incidence curves were presented for the two HF therapies overall and additionally by LVEF < 40% and ≥ 40%.

Detailed adjusted Cox regression analyses comparing specific subgroups of the modern (with and without ARNI and SGLT2i) and conventional therapies (with and without MRA) were performed and presented in a forest plot.

All tests were two-tailed and *p*-values < 0.05 were considered statistically significant. All analyses were performed using SAS® Software v9.4 (SAS Institute Inc., Cary, NC, USA).

## Results

### Patient population and baseline characteristics

Some 20,849 patients were identified, of whom 20,140 were included in the conventional treatment group and 709 in the modern treatment group. As shown in Table 1, compared to patients on conventional treatment, patients in the modern treatment group were more often of the male sex (77% vs. 62%), younger (40% vs. 63% ≥ 70 years old), more symptomatic (40% vs. 33% in NYHA class III-IV), lower LVEF (51% vs. 28% LVEF < 30%), and higher eGFR (75% vs. 67% eGFR ≥ 60 ml/min/1.73 m^2^). A similar pattern was observed between conventional and modern treatment groups, regardless of EF, sex, age, and eGFR. However, clinical characteristics differed in patients in the modern treatment group due to different EF, age, sex, and eGFR categories. For example, compared to EF ≥ 40% in modern treatment, those in EF < 40% who received modern HF therapy were younger (mean 64.2 vs. 69.1 years), more often male (77% vs. 73%), more symptomatic (42% vs. 36% in NYHA class III-IV), higher LVEF (60% vs. 68% LVEF < 30%), higher NT-proBNP (median 1972 vs. 1493 pg/ml), higher eGFR (75% vs. 71% eGFR ≥ 60), more device (ICD, CRT), and more ARNI (67% vs. 27%) (Supplementary Table 2).

**Table 1:** Patient demographics, clinical data, and comorbidities at the index visit in the SwedeHF comparing modern vs. conventional HF therapy: overall cohort and propensity score 1:1 matched cohort excluding ARNI in the matching procedure

Variable	Overall cohort			Propensity score 1:1 matched cohort		
	Conventional, *N* = 20,140	Modern, *N* = 709	*P*-value	Conventional, *N* = 707	Modern, *N* = 707	*P*-value
Patient demographics						
Male sex	12,584 (62.5%)	543 (76.6%)	< .0001	542 (76.7%)	541 (76.5%)	1.00
Age at SWEDEHF index visit	72.3 ± 12.3 74 (18–104)	65.0 ± 12.1 66 (23–91)	< .0001	65.4 ± 12.9 66 (24–98)	65.0 ± 12.1 66 (23–91)	0.57
Age at admission			< .0001			0.45
< 70 years	7529 (37.4%)	423 (59.7%)		436 (61.7%)	421 (59.5%)	
≥ 70 years	12,611 (62.6%)	286 (40.3%)		271 (38.3%)	286 (40.5%)	
Body mass index (kg/m^2^)			< .0001			0.98
< 18.5	382 (2.4%)	7 (1.1%)		6 (1.0%)	7 (1.1%)	
18.5–25	5577 (34.4%)	145 (23.2%)		146 (23.9%)	144 (23.2%)	
> 25–30	5914 (36.5%)	224 (35.9%)		213 (34.8%)	223 (35.9%)	
> 30–35	2814 (17.4%)	148 (23.7%)		148 (24.2%)	148 (23.8%)	
> 35	1522 (9.4%)	100 (16.0%)		99 (16.2%)	100 (16.1%)	
Systolic blood pressure (mmHg)	128.7 ± 21.0 128 (10–240)	120.9 ± 19.8 120 (12–198)	< .0001	127.8 ± 21.8 125 (76–240)	120.9 ± 19.9 120 (12–198)	< .0001
Diastolic blood pressure (mmHg)	75.4 ± 12.3 75 (35–180) *n* = 13,119	73.8 ± 11.3 73 (40–120) *n* = 554	0.0070	75.5 ± 13.7 75 (40–180) *n* = 544	73.9 ± 11.3 73 (40–120) *n* = 552	0.08
Heart rate (bpm)	73.2 ± 16.0 70 (30–170) *n* = 13,060	74.5 ± 15.0 73 (42–140) *n* = 550	0.0074	73.9 ± 15.6 72 (42–137) *n* = 540	74.5 ± 15.0 73 (42–140) *n* = 548	0.37
NYHA functional class			< .0001			0.75
I	1862 (12.9%)	59 (9.8%)		57 (9.6%)	59 (9.8%)	
II	7810 (54.3%)	302 (49.9%)		304 (51.4%)	302 (50.1%)	
III	4515 (31.4%)	235 (38.8%)		222 (37.6%)	233 (38.6%)	
IV	194 (1.3%)	9 (1.5%)		8 (1.4%)	9 (1.5%)	
LVEF (%)			< .0001			0.73
≥ 50%	3108 (16.4%)	36 (5.1%)		37 (5.2%)	36 (5.2%)	
40– < 50%	4591 (24.2%)	77 (11.0%)		81 (11.5%)	77 (11.0%)	
30– < 40%	6038 (31.8%)	233 (33.3%)		238 (33.8%)	233 (33.4%)	
< 30%	5234 (27.6%)	354 (50.6%)		349 (49.5%)	352 (50.4%)	
Potassium (mmol/l)	4.25 ± 0.41 4.2 (2.2–6.5) *n* = 12,948	4.32 ± 0.42 4.3 (3.2–5.8)\ *n* = 548	< .0001	4.30 ± 0.41 4.3 (3.0–5.7) *n* = 543	4.32 ± 0.42 4.3 (3.2–5.8) *n* = 546	0.19
NT-proBNP (pg/ml)	4365.6 ± 6368.5 2325 (1–111,601) *n* = 15,027	3088.2 ± 3753.3 1934 (50–32,365) *n* = 600	< .0001	3519.6 ± 5906.3 1846 (24–62,878) *n* = 592	3091.0 ± 3759.2 1934 (50–32,365) *n* = 598	0.93
NT-proBNP cat. (pg/ml)			< .0001			0.80
< = 900	3302 (22.0%)	166 (27.7%)		163 (27.5%)	166 (27.8%)	
> 900–2500	4578 (30.5%)	194 (32.3%)		200 (33.8%)	193 (32.3%)	
> 2500–5000	3389 (22.6%)	134 (22.3%)		127 (21.5%)	133 (22.2%)	
> 5000	3758 (25.0%)	106 (17.7%)		102 (17.2%)	106 (17.7%)	
eGFR (CKD-EPI)	69.2 ± 21.1 70 (3–285) *n* = 13,005	74.8 ± 20.7 77 (20–142) *n* = 549	< .0001	73.2 ± 22.5 75 (7–138) *n* = 543	74.7 ± 20.7 76 (20–142) *n* = 547	0.47
eGFR (CKD-EPI, ml/min/1.73 m^2^)			< .0001			0.14
< 60	4332 (33.3%)	139 (25.3%)		160 (29.5%)	139 (25.4%)	
≥ 60	8673 (66.7%)	410 (74.7%)		383 (70.5%)	408 (74.6%)	
Medical history at the index visit						
IHD	8423 (41.8%)	353 (49.8%)	< .0001	358 (50.6%)	351 (49.6%)	0.75
Valve disease or surgery	2797 (13.9%)	53 (7.5%)	< .0001	63 (8.9%)	53 (7.5%)	0.38
Hypertension	13,555 (67.3%)	506 (71.4%)	0.025	507 (71.7%)	504 (71.3%)	0.91
Atrial fibrillation	10,427 (51.8%)	279 (39.4%)	< .0001	260 (36.8%)	279 (39.5%)	0.32
Chronic obstructive pulmonary disease	3123 (15.5%)	95 (13.4%)	0.14	105 (14.9%)	95 (13.4%)	0.49
Diabetes mellitus	4567 (22.7%)	419 (59.1%)	< .0001	432 (61.1%)	417 (59.0%)	0.45
Blood diseases	3576 (17.8%)	120 (16.9%)	0.62	122 (17.3%)	118 (16.7%)	0.83
Stroke/TIA	2464 (12.2%)	82 (11.6%)	0.64	82 (11.6%)	81 (11.5%)	1.00
Psychiatric diagnoses in the past 3 years before admission	2856 (14.2%)	118 (16.6%)	0.07	132 (18.7%)	117 (16.5%)	0.33
Musculoskeletal diseases in the past 3 years before admission	3479 (17.3%)	94 (13.3%)	0.0045	94 (13.3%)	94 (13.3%)	1.00
Malignant cancer in the past 3 years before admission	2098 (10.4%)	60 (8.5%)	0.10	60 (8.5%)	60 (8.5%)	1.00
ICD	373 (1.9%)	44 (6.2%)	< .0001	40 (5.7%)	42 (6.0%)	0.91
CRT	198 (1.0%)	18 (2.5%)	0.0005	14 (2.0%)	17 (2.4%)	0.72
Medications						
ARNI dispensed 3 m before to 6 m after index	0 (0.0%)	430 (60.6%)	< .0001	0 (0.0%)	428 (60.5%)	< .0001
ACEi/ARB dispensed 3 m before to 6 m after index	20,140 (100.0%)	375 (52.9%)	< .0001	707 (100.0%)	374 (52.9%)	< .0001
SGLT2 inhibitors dispensed 3 m before to 6 m after index	0 (0.0%)	418 (59.0%)	< .0001	0 (0.0%)	416 (58.8%)	< .0001
Diuretics	10729 (71.8%)	523 (75.1%)	0.052	370 (75.4%)	521 (75.1%)	0.95

*AF* atrial fibrillation, *HF* heart failure, *SwedeHF* Swedish heart failure registry, *NYHA* New York heart association, *LVEF* left ventricular ejection fraction, *eGFR* estimated glomerular filtration rate, *CKD-EPI* chronic kidney disease epidemiology collaboration, *TIA* transient ischemic attack, *ICD* implantable cardioverter-defibrillator, *CRT* cardiac resynchronization therapy, *ARNI* angiotensin receptor-neprilysin inhibitor, *ACEi* angiotensin-converting enzyme inhibitor, *ARB* angiotensin receptor blocker, *SGLT2* sodium-glucose transport protein 2, *NT-proBNP* N-terminal pro-B-type natriuretic peptide, *IHD* Ischemic Heart Disease, *m* month

Data are presented as mean ± standard deviation, median (range), and number of observations or number (percentage)

For test between two groups with respect to dichotomous variables, Fisher’s exact test was used; for ordered categorical variables, the Mantel–Haenszel chi-square trend test was calculated; and for continuous variables, the Mann–Whitney *U*-test was applied

Propensity score matching is performed that included age, sex, BMI, NYHA, LVEF, NT-proBNP, eGFR, IHD, valve surgery, hypertension, AF, diabetes, ICD, and CRT

Likewise, compared to men on modern treatment, women who received modern HF therapy were older (67.2 vs. 64.3 years) and had a higher proportion of NYHA class III-IV (48% vs. 38%), higher LVEF (44% vs. 53% LVEF < 30%), higher NT-proBNP (median 2607 vs. 1841 pg/ml), and lower eGFR (59% vs. 79% eGFR ≥ 60 ml/min/1.73 m^2^) (data not shown). In addition, those aged ≥ 70 years in the modern therapy group were less often male (74% vs. 78%) and had a higher proportion of NYHA class III-IV (45% vs. 37%), higher LVEF (41% vs. 57% LVEF < 30%), higher NT-proBNP (median 2180 vs. 1681 pg/ml), and lower eGFR (59% vs. 85% eGFR ≥ 60 ml/min) (data not shown).

Following propensity score 1:1 matching with and without the inclusion of ARNI in the matching procedure, balanced treatment groups were achieved for patient characteristics and comorbidity status (Table 1, Supplementary Table 3).

### All-cause and cardiovascular mortality: overall HF cohort and propensity-matched cohort

**Fig. 1:**
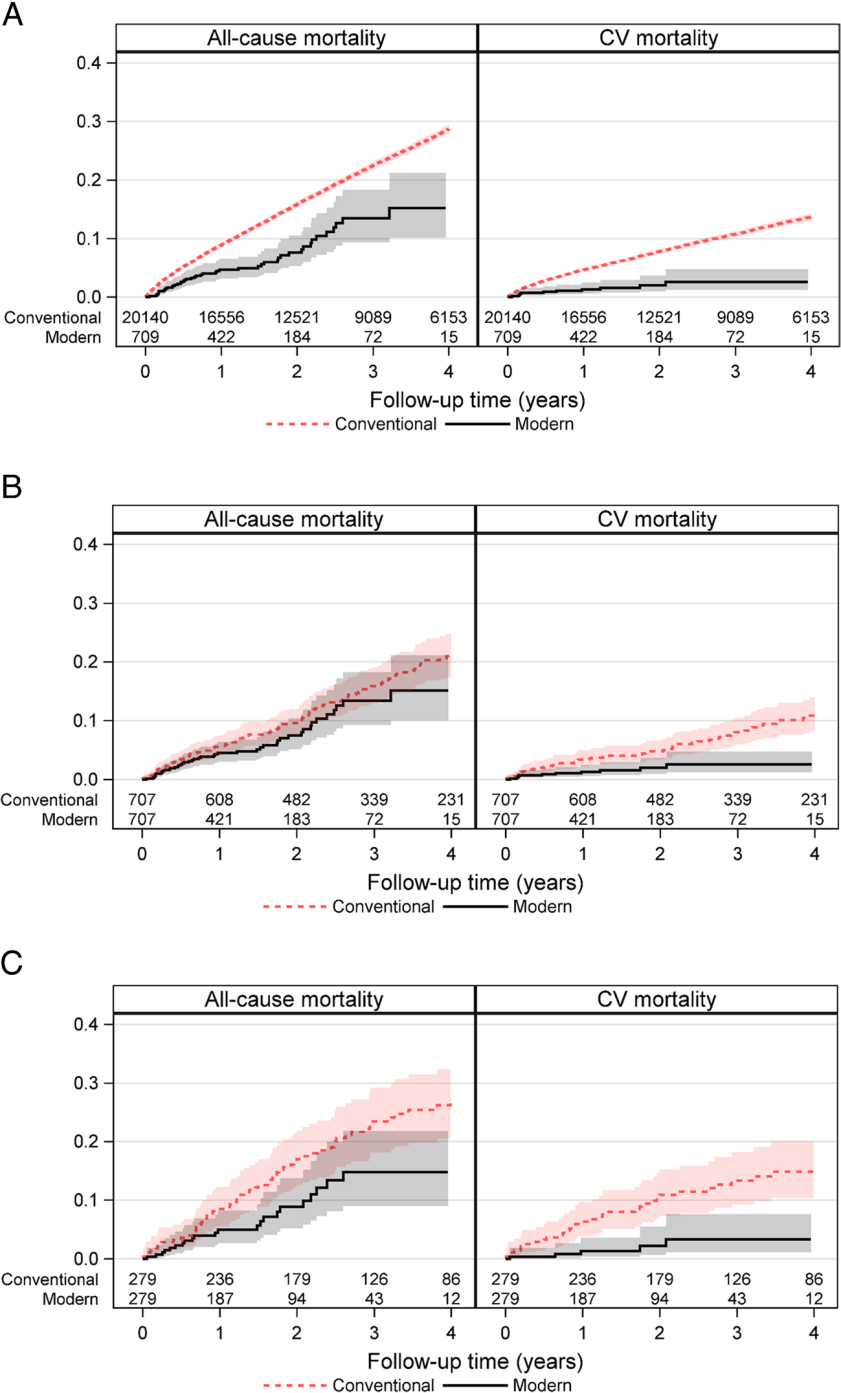
Cumulative incidence curve for conventional vs. modern HF therapy (**A** overall cohort, **B** propensity score 1:1 matched groups excluding ARNI, **C** 1:1 propensity score 1:1 matched groups including ARNI in the matching procedure). Abbreviations: CV, cardiovascular; ARNI, angiotensin receptor-neprilysin inhibitor; HR, hazard ratio

**Fig. 2:**
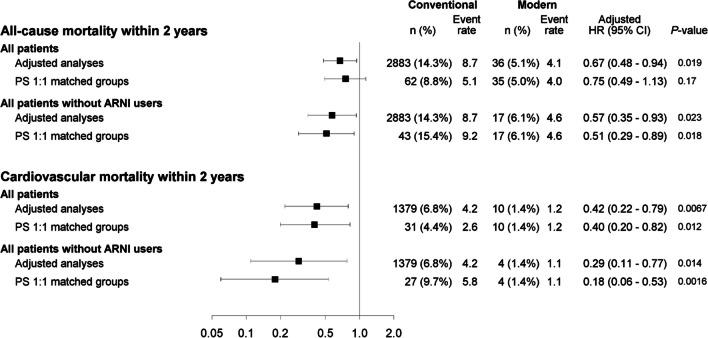
Adjusted Cox regression analyses for all-cause and cardiovascular mortality within 2 years from the index visit performed on the overall cohort and 1:1 propensity score-matched groups, with and without including patients on ARNI. Abbreviations: ARNI, angiotensin receptor-neprilysin inhibitor; HR, hazard ratio; CI, confidence interval

In the overall HF cohort, all-cause mortality occurred in 5225/20,140 (25.9%) patients with conventional HF treatment. In contrast, it developed in only 47/709 (6.6%) patients in the modern HF therapy group. Within 2 years of index registration, all-cause mortality was seen in 14.3% of patients in the conventional HF therapy group versus 5.1% in the modern HF treatment group. Modern HF therapy was associated with a significant 28% reduction in all-cause mortality compared to conventional HF treatment (adjusted HR [aHR], 0.72 (95% CI 0.54–0.96); *p* = 0.024) and had 33% reduced all-cause mortality (aHR, 0.67 (95% CI 0.48–0.94); *p* = 0.019) within 2 years of index registration (Table 2, Fig. 1A). Similarly, modern HF therapy was associated with a significant 62% reduction in CV mortality compared to conventional HF treatment (aHR, 0.38 (95% CI 0.21–0.68); *p* = 0.0013). In addition, we found a 58% reduced CV mortality (aHR, 0.42 (95% CI 0.22–0.79); *p* = 0.0067) within 2 years of index registration (Table 2, Figs. 1A and 2).

**Table 2:** Event rates, number of events, follow-up time, age and sex-adjusted, and fully adjusted HRs (Cox regression) comparing modern to conventional HF therapy (overall cohort, propensity score 1:1 matched groups exclusive and inclusive ARNI)

Endpoint	Conventional			Modern			Modern vs. conventional
	*n*/*N* (%) events No. events/follow-up time	Follow-up time, Median (IQR)	Event rate (95% CI) per 100 person-years	*n* (%) events No. events/Follow-up time	Follow-up time Median (IQR)	Event rate (95% CI) per 100 person-years	*Hazard ratio (95% CI), *P*-value
Overall cohort							
All-cause mortality	5225/20,140 (25.9%)	2.72 (1.34–4.54) Sum = 60,911	8.6 (8.3–8.8)	47/709 (6.6%)	1.23 (0.64–2.04) Sum = 1035	4.5 (3.3–6.0)	0.72 (0.54–0.96) *p* = 0.024
All-cause mortality within 2 y	2883/20,140 (14.3%)	2.00 (1.34–2.00) Sum = 32,979	8.7 (8.4–9.1)	36/709 (5.1%)	1.23 (0.64–2.00) Sum = 868	4.1 (2.9–5.7)	0.67 (0.48–0.94) *p* = 0.019
Cardiovascular mortality	2218/20,140 (11.0%)	2.72 (1.34–4.54) Sum = 60,911	3.6 (3.5–3.8)	11/709 (1.6%)	1.23 (0.64–2.04) Sum = 1035	1.1 (0.5–1.9)	0.38 (0.21–0.68) *p* = 0.0013
Cardiovascular mortality within 2 y	1379/20,140 (6.8%)	2.00 (1.34–2.00) Sum = 32,979	4.2 (4.0–4.4)	10/709 (1.4%)	1.23 (0.64–2.00) Sum = 868	1.2 (0.6–2.1)	0.42 (0.22–0.79) *p* = 0.0067
Propensity score 1:1 matched groups excluding ARNI in the matching process							
All-cause mortality	135/70 (19.1%)	2.86 (1.65–4.82) Sum = 2268	6.0 (5.0–7.0)	46/707 (6.5%)	1.23 (0.64–2.04) Sum = 1032	4.5 (3.3–5.9)	0.79 (0.55–1.12) *p* = 0.18
All-cause mortality within 2 y	62/707 (8.8%)	2.00 (1.65–2.00) Sum = 1205	5.1 (3.9–6.6)	35/707 (5.0%)	1.23 (0.64–2.00) Sum = 866	4.0 (2.8–5.6)	0.75 (0.49–1.13) *p* = 0.17
Cardiovascular mortality	63/707 (8.9%)	2.86 (1.65–4.82) Sum = 2268	2.8 (2.1–3.6)	11/707 (1.6%)	1.23 (0.64–2.04) Sum = 1032	1.1 (0.5–1.9)	0.35 (0.18–0.68) *p* = 0.0020
Cardiovascular mortality within 2 y	31/707 (4.4%)	2.00 (1.65–2.00) Sum = 1205	2.6 (1.7–3.7)	10/707 (1.4%)	1.23 (0.64–2.00) Sum = 866	1.2 (0.6–2.1)	0.40 (0.20–0.82) *p* = 0.012
Propensity score 1:1 matched groups including ARNI in the matching							
All-cause mortality	67/279 (24.0%)	2.82 (1.46–4.47) Sum = 848	7.9 (6.1–10.0)	23/279 (8.2%)	1.39 (0.85–2.39) Sum = 472	4.9 (3.1–7.3)	0.58 (0.36–0.94) *p* = 0.028
All-cause mortality within 2 y	43/279 (15.4%)	2.00 (1.46–2.00) Sum = 467	9.2 (6.7–12.4)	17/279 (6.1%)	1.39 (0.85–2.00) Sum = 373	4.6 (2.7–7.3)	0.51 (0.29–0.89) *p* = 0.018
Cardiovascular mortality	35/279 (12.5%)	2.82 (1.46–4.47) Sum = 848	4.1 (2.9–5.7)	5/279 (1.8%)	1.39 (0.85–2.39) Sum = 472	1.1 (0.3–2.5)	0.21 (0.08–0.53) *p* = 0.0011
Cardiovascular mortality within 2 y	27/279 (9.7%)	2.00 (1.46–2.00) Sum = 467	5.8 (3.8–8.4)	4/279 (1.4%)	1.39 (0.85–2.00) Sum = 373	1.1 (0.3–2.7)	0.18 (0.06–0.53) *p* = 0.0016

*BMI* body mass index, *HF* heart failure, *SwedeHF* Swedish heart failure registry, *NYHA* New York heart association, *LVEF* left ventricular ejection fraction, *eGFR* estimated glomerular filtration rate, *ICD* implantable cardioverter-defibrillator, *CRT* cardiac resynchronization therapy, *ARNI* angiotensin receptor-neprilysin inhibitor, *NT-proBNP* N-terminal pro-B-type natriuretic peptide, *IHD* Ischemic Heart Disease, *y* year, *CI* confidence interval, *HR* hazard ratio, *IQR* interquartile range

Confidence interval for unadjusted event rates per 100 person-years is obtained from exact Poisson confidence limits

Cox regression was used for time to any event presented by HR

Adjusted for age, sex, BMI, NYHA, LVEF, NT-proBNP, eGFR, IHD, any valve surgery or disease, hypertension, atrial fibrillation, diabetes, ICD, CRT

In the propensity 1:1 matched cohort in which ARNI was excluded in the matching procedure, all-cause mortality was observed in 135/707 (19.1%) patients with conventional treatment and 46/707 (6.5%) with modern HF therapy associated with a 21% non-statistically significant risk reduction (in all-cause mortality in the modern HF treatment group compared to those treated conventionally (aHR, 0.79 (95% CI 0.55–1.12); *p* = 0.18) and a 25% non-significant risk reduction (HR, 0.75 (95% CI 0.49–1.13); *p* = 0.17) within 2 years of index registration. However, in the same propensity score 1:1 matched cohort, a significantly reduced CV mortality of 65% was seen in patients who received modern HF treatment compared to those given conventional HF treatment (aHR, 0.35 (95% CI 0.18–0.68); *p* = 0.0020). Similarly, modern HF therapy was associated with a significant risk reduction in CV mortality of 60% within 2 years of index registration (aHR, 0.40 (95% CI 0.20–0.82); *p* = 0.012). (Table 2, Figs. 1B and 2).

MRA our results were associated with a statistically significant risk reduction of 42% in all-cause mortality in the modern HF treatment group compared to the conventional treatment group (aHR, 0.58 (95% CI 0.36–0.94); *p* = 0.028) and a 49% (aHR, 0.51 (95% CI 0.29–0.89); *p* = 0.018) risk reduction within 2 years of index registration. Likewise, a statistically significant reduced CV mortality of 79% was noted in patients in the modern HF treatment group compared to those in the conventional HF treatment group (aHR, 0.21 (95% CI 0.08–0.53); *p* = 0.0011) and 82% within 2 years of index registration (aHR, 0.18 (95% CI 0.06–0.53); *p* = 0.0016) (Table 2, Figs. 1C and 2).

### Comparison of components within modern HF therapy

**Fig. 3:**
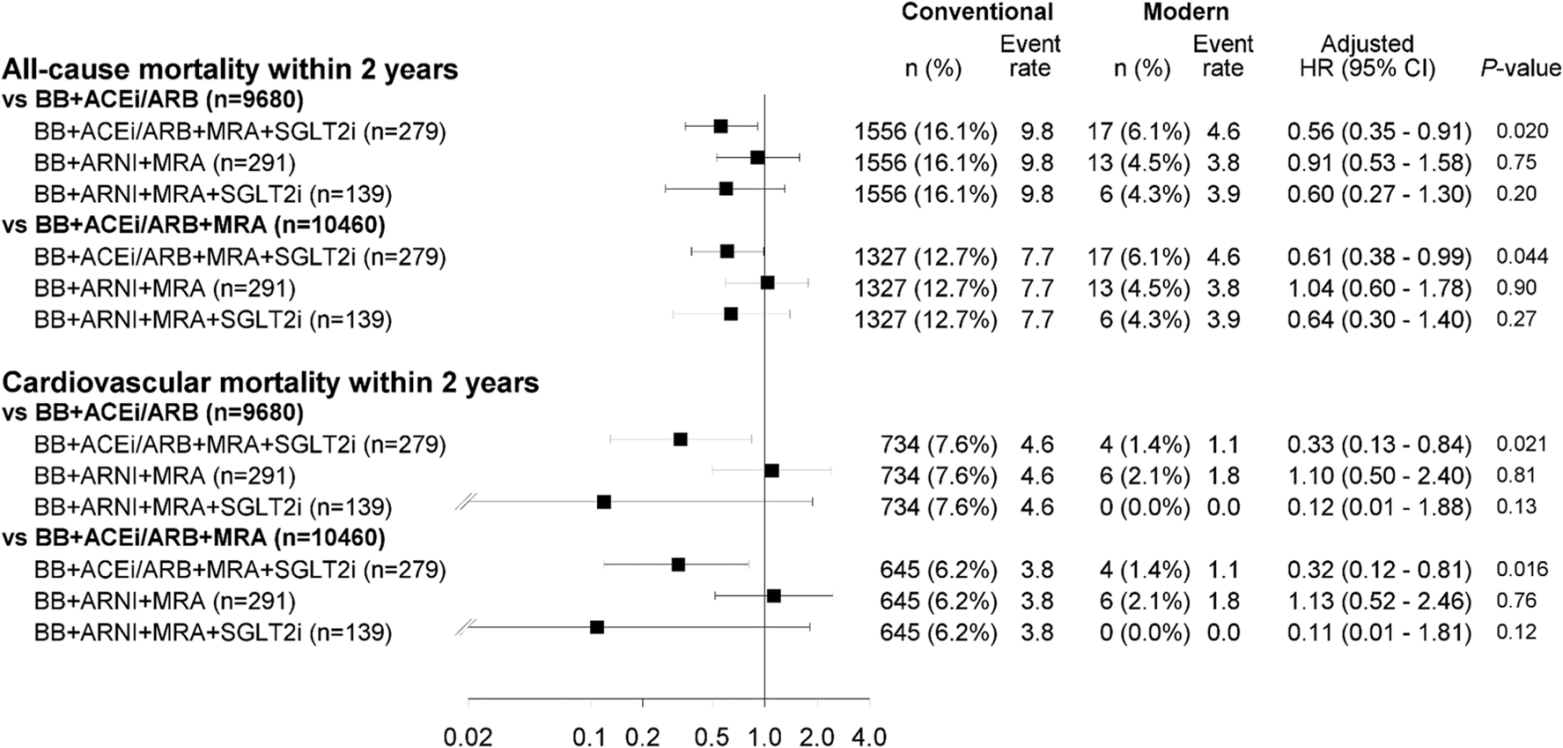
Adjusted Cox regression analyses for all-cause and cardiovascular mortality within 2 years from the index visit comparing different subgroups by modern vs. conventional treatment (complete cohort). Abbreviations: ARNI, angiotensin receptor-neprilysin inhibitor; *ACEi* angiotensin-converting enzyme inhibitor; ARB, angiotensin receptor blocker; BB, beta-blocker; CI, confidence interval; HR, hazard ratio; MRA, mineralocorticoid receptor antagonist; SGLT2i, sodium-glucose cotransporter 2 inhibitor

Adjusted Cox regression analyses for all-cause and CV mortality within 2 years from the index visit were performed to compare different subgroups of modern vs. conventional treatment in the overall cohort. All subgroups containing SGLT2i, or both ARNI and SGLT2i, in addition to BB and MRA, were significantly or numerically superior to those only treated without SGLT2i for risk reduction associated with mortality, regardless of all-cause or CV mortality (Fig. 3).

### Interactions between conventional and modern HF treatment and different subgroups on all-cause and CV mortality

**Fig. 4:**
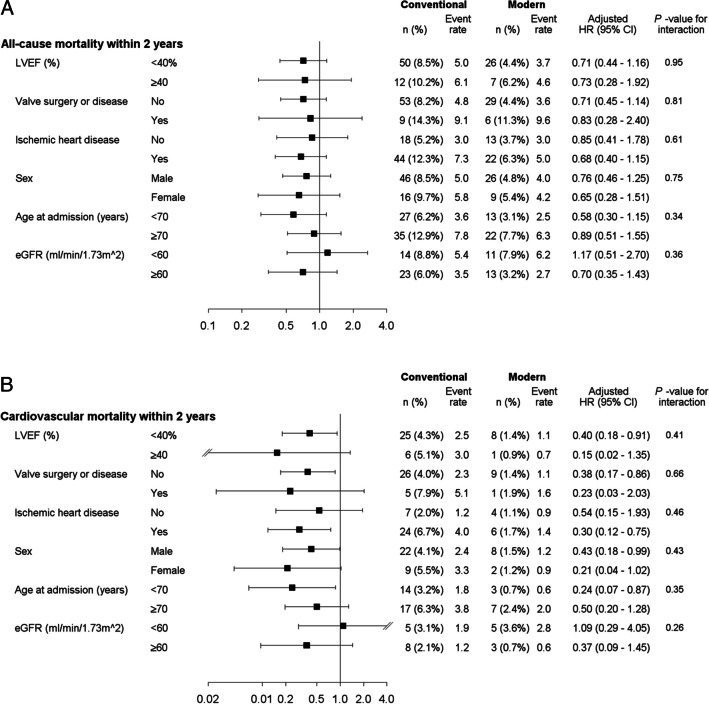
Adjusted Cox regression interaction analyses for outcome within 2 years from the index visit for propensity score 1:1 matched groups excluding ARNI (A all-cause mortality, B cardiovascular mortality). Abbreviations: SwedeHF, Swedish heart failure registry; LVEF, left ventricular ejection fraction; eGFR, estimated glomerular filtration rate; ARNI, angiotensin receptor-neprilysin inhibitor; HR, hazard ratio; CI, confidence interval

Associations related to modern HF therapy and its interactions with different subgroups on all-cause and CV mortality were assessed for EF (< 40% and ≥ 40%), sex, age (< 70 and ≥ 70 years), eGFR (< 60 and ≥ 60 ml/min/1.73 m^2^), and etiology of HF (Fig. 4). No significant interactions were observed for all subgroups, regardless of the studied outcome variable.

## Discussion

This study demonstrates that modern HF therapy in a real-world nationwide Swedish HF population is associated with a statistically significant risk reduction in all-cause and CV mortality. To our knowledge, this is one of the few studies reporting the real-world comparison of the efficacy of conventional vs. modern HF therapy across the EF spectrum. During the past decades, HF guidelines have been based on three fundamental therapies: ACEi/ARB, BB, and MRA on the chronological sequence of the trials. Today, novel agents (ARNI and SGLT2i) and standard therapies (BB + ACEi/ARB + MRA) have further reduced the risk of the primary endpoint of CV mortality or hospitalization in patients with HFrEF. Therefore, current guidelines recommend four fundamental treatments: ACEi/ARB/ARNI, BB, MRA, and SGLT2i in combination as the default strategy for HFrEF. However, these pharmacological agents were previously studied separately in a placebo-controlled randomized trial with a different background therapy and unique study design. This approach made uncertain the expected overall treatment benefits from several therapeutic agents in a combined regimen. Moreover, a direct comparison between conventional and modern treatment strategies is largely lacking, not to mention real-world data comparing the effectiveness of these two strategy-based therapies.

Because we have access to the SwedeHF, we could demonstrate that modern HF therapy was associated with a significant 28% reduction in all-cause mortality and a 62% reduction in CV mortality compared to conventional HF treatment. This finding was supported by a propensity-matched cohort in which a significantly reduced CV mortality of 65% was seen in patients who received modern HF treatment compared to those treated conventionally. Associations with effects on all-cause and CV mortality were consistent in all subgroups, regardless of EF < 40% or ≥ 40%, sex, age < 70 or ≥ 70 years, eGFR < 60 or ≥ 60 ml/min, and etiology of HF.

Our results are clinically relevant. First, we assess the association of effect with standard vs. modern HF therapy in all HF patients, regardless of EF, age, sex, eGFR, and etiology. Current HF guidelines give class IA recommendations of four fundamental treatments (ACEi/ ARB/ARNI, BB, MRA, and SGLT2i) as the default strategy for HFrEF. However, growing evidence suggests that ACEi/ARB, BB, MRA, and ARNI may also be considered in HFmrEF (class IIb recommendations), as they may reduce the risk of HF hospitalization and/or mortality [9]. Our findings support this rather weak recommendation. Second, our patients are from a real-world setting without any exclusions. This point is particularly relevant, as all RCTs are highly selective with many exclusion criteria. Among them, eGFR < 30 ml/min/1.73 m^2^ is a commonly used exclusion criterion in most studies, except in the EMPEROR-Reduced trial, where eGFR < 20 ml/min/1.73 m^2^ was applied. Third, for the best comparison, the conventional treatment group was required to be treated with both fundamental agents (BB and at least one of the ACEi/ARB medications) with or without MRA. This treatment regimen is unique to our study. As foundations for conventional HF therapy, BB was prescribed only in 10% of the Randomized Aldactone Evaluation Study (RALES) with spironolactone [17] and 8.3% in Studies of the Left Ventricular Dysfunction (SOLVD) trial with enalapril [18]. Even in a later study, the Eplerenone in Mild Patients Hospitalization and Survival Study in Heart Failure (EMPHASIS-HF), BB was used in 87% and ACEi/ARB in 94% [19].

Moreover, in our study, MRA is one of the obligatory treatments in modern HF therapy as a foundation for modern HF therapy with novel agents such as ARNI or SGLT2i, which were used in addition to background therapy, including BB and MRA. However, MRA was used only in 71.5% of the study patients in the DAPA-HF [7], 70.1% in the EMPEROR-Reduced [8], and 54.2% in the PARADIGM-HF trial [6]. Therefore, our study has added new data from the real world about strategy-based effectiveness for conventional and modern HF therapy. It is noteworthy to mention that the focus of this work is not to assess effect from a single treatment but the aggregated effect from a therapeutic regime (conventional vs modern). For instance, our data do not provide information on whether beta-blocker is more beneficial than others.

Using real-world data without exclusions, we observed a lower proportion of CV mortality (nearly 40%) in all-cause mortality. In our study, the event rate of all-cause mortality was 8.6 per 100 person-years and only 3.6 for CV mortality. However, all landmark HF trials with modern agents showed a high proportion of CV mortality. For instance, in the DAPA-HF event, all-cause and CV mortality rates were 9.5 and 7.9 per 100 person-years, respectively [7]. Similarly, corresponding event rates of all-cause and CV mortality were 10.7 and 8.1 per 100 person-years in the EMPEROR-REDUCED trial [8]. All-cause and CV mortality were 19.8% and 16.5%, respectively, in the PARADIGM-HF trial [6]. These findings confirm that study cohorts from RCT trials have a different risk profile than a real-world cohort despite the same type of HF patients and highlight that our real-world data complement existing data from RCT trials.

Our data have also shown no significant interactions observed with different categories of LVEF. This is clinically relevant. Hitherto most of GDMT except SGLT2i have weaker recommendations in HFmrEF and HFpEF. But based on our data, it appears that modern therapy as a whole is likely equally effective in EF > 40% as in EF ≤ 40%. However, this needs to be further studied in a randomized controlled trial.

We know our study can only determine associations and cannot infer efficacy. Therefore, we cannot compare the magnitude of risk reduction in our study with available data. Nevertheless, our results align with a meta-analysis in which ARNI + BB + MRA, SGLT2i + ACEi + BB + MRA, and ivabradine + ACEi + BB + MRA were the most effective treatment combinations [20]. In addition, in a cross-trial analysis, Vaduganathan et al. compared the treatment effects of ARNI, BB, MRA, and SGLT2 inhibitor with ACE inhibitor/ARB and BB in chronic HFrEF derived from the EMPHASIS-HF, PARADIGM-HF, and DAPA-HF trials. The authors found that the anticipated aggregate treatment effects of former combined comprehensive therapy are substantial [14]. Similar results were reported in a systematic network meta-analysis of a series of RCTs, suggesting that the estimated aggregate benefit is greatest for a combination of ARNi, BB, MRA, and SGLT2i [21]. All these analyses are based on data derived from trials with selection bias. Interestingly, despite our data were extracted from a time (2003–2020) when GDMT has traditionally been implemented in a stepwise order, our results from the real world provide supporting evidence that a combination of SGLT2i and ARNI is superior to conventional treatment. In the meantime, our data from adjusted Cox regression analyses showed that all subgroups containing SGLT2i, or both ARNI and SGLT2i, in addition to BB and MRA, were significantly or numerically superior to those only treated without SGLT2i for risk reduction associated with mortality, both all-cause and CV mortality. There seems to be a trend of the superiority of the combination of SGLT2i with ACEi/ARB instead of ARNI.

Our study was intended to investigate not only individual treatment agents but also treatment regimens. Still, we tried to compare two novel agents in modern HF therapy. We adopted two propensity score models with 1:1 matching: one excluded ARNI and the other included ARNI in the matching procedure (i.e., no ARNI was used in the two treatment groups). We observed statistically significant risk reductions of 42% in all-cause mortality and 79% in CV mortality in the modern HF treatment group compared to those treated conventionally. In other words, modern therapy containing SGLT2i, or both ARNI and SGLT2i, is significantly or numerically superior to those treated without SGLT2i for risk reduction in mortality, regardless of all-cause or CV mortality. However, our data must be interpreted cautiously as individuals treated with SGLT2i may differ from those treated with ARNI. This is because our database was collected up to 2020. Most of the prescriptions of SGLT2i probably occurred in patients with HF and type 2 diabetes mellitus (T2DM), as SGLT2i was initially approved for the treatment of T2DM in 2012 and the first landmark HF trial with SGLT2i was published in 2019. The event rate was higher in patients with HF with coexisting DM. Thus, a more pronounced treatment benefit was expected.

The year 2021 is a turning point in HF guideline recommendation from sequential order to early or simultaneous initiation (ACEI/ARB/ARNI, BB, MRA, and SGLT2i) within 2 weeks. But our data were extracted from a time (2003–2020) when GDMT has traditionally been implemented in the consecutive order by which they were added stepwise to the guidelines, e.g., starting ACEi/ARB + BB, followed by the addition of MRA. MRA has been added-on drug since 2008 through 2016 until 2021. In the meantime, ARNI has also been recommended in Sweden as added-on drug to replace ACEI/ARB, and SGLT2i was used mainly due to T2DM. Therefore, in order to reflect clinical practice in the real world, together with the availability of our database, conventional therapy was defined as having obligatory dual treatments with BB, ACEi/ARB as the backbone and with or without MRA as an option, whereas modern therapy must meet 3 criteria: (1) having obligatory at least triple treatments with BB, ACEi/ARB/ARNI, and MRA as the backbone; (2) containing MRA; and (3) containing either SGLT2i or ARNI or both. Six months after index registration was necessary because triple treatment is required to assess the additional effects of novel agents.

## Strengths and limitations

The strength of this study is that we have access to several large, high-quality national registries linked to each other, making it possible to identify clinical data, data on drug dispensation, hospital readmissions, and mortality.

One major limitation is the small sample size of those treated with ARNI or SGLT2i novel agents, preventing subgroup analysis for EF 41–49% and EF > 50%. Another limitation is that we could not verify the diagnosis individually. However, because SGLT2i was initially approved to treat T2DM in 2012, T2DM was likely the most common diagnosis in this patient cohort. Still, off-label use in type 1 DM could also occur in those with a low risk of ketoacidosis. This possibility should not affect our results, though. On the contrary, it can strengthen our finding that SGLT2i is effective in patients with HF, regardless of EF and type of DM. Because of the coverage restriction of the SwedeHF, not all patients with HF-initiated SGLT2i were reported to the SwedeHF. To address this issue, we limited the inclusion of patients to those who received SGLT2i < 3 months before and 6 months after the index date. In addition, as in many studies, we cannot be sure that patients adhered to medication regimens throughout the study. Although the SwedeHF collects data from a large number of variables, allowing us to perform extensive adjustments, we cannot rule out the presence of residual and unmeasured confounding. As mentioned, observational studies can only establish that significant associations exist between predictor and outcome variables, not causal relationships. Previous studies have also shown that less ill patients, more males, and younger and better-treated patients are enrolled in the SwedeHF, limiting the generalizability of our results [22]. Because earlier indications of SGLT2i were only in diabetes mellitus, the study population for modern therapy is predominately patients with underlying diabetes mellitus, which is not representative of all etiologies in HF. However, diabetes is still one of the most common causes, and in DAPA-HF and EMPEROR-REDUCED trials, diabetes is as common as 42% and 50%, respectively.

Indeed, the recommendation class and evidence level of GDMT (Guideline directed medical therapy) are different between EF ≤ and > 40%. But those recommended for EF ≤ 40% can be considered in EF > 40% despite weaker indication (IIb) and limited evidence except SGLT2i is strongly recommended regardless of EF which was however issued recently after completion of this study. In other words, our data extracted from the time period 2013–2020 are not allowed to study GDMT in HFpEF, as evidence-based effective GDMT for HFpEF is not available until 2021. In our daily clinical practice, we do our best to treat HFpEF empirically. Since our study is based primarily on real-world clinical data, we prefer to present data as they are. Nevertheless, we have performed interaction analysis which showed no significant interactions, suggesting that modern therapy as a whole is likely equally effective in EF > 40% as in EF ≤ 40%. This should be regarded as an important observation. Another important is the small sample size of those treated with ARNI or SGLT2i as a necessary part of modern therapy, which did not allow further differentiating different EF. Nevertheless, it is unlikely that the observed effect was driven by other factors than GDMT as our study is aimed to compare two treatment strategies, and all other factors were adjusted.

Last but not least, since SwedeHF did not achieve 100% coverage of patients with heart failure in Sweden currently, there are no data about these patients who were not reported to SwedeHF whether they received similar treatments or not. Accordingly, the conclusion from this study may not be representative of those who are not registered in SwedeHF.

## Conclusion

Our study demonstrates that modern HF therapy in a real-world nationwide Swedish HF population is associated with a statistically significant risk reduction in all-cause and CV mortality, regardless of EF, age, sex, renal function, and HF etiology.

## Supplementary Information

Below is the link to the electronic supplementary material.

Supplementary file1 (DOCX 98 KB)

Supplementary file2 (DOCX 411 KB)

Supplementary file3 (DOCX 15 KB)

Supplementary file4 (DOCX 23 KB)

Supplementary file5 (DOCX 19 KB)
