# Supplementary figures and images for: Modern heart failure treatment is superior to conventional treatment across the left ventricular ejection spectrum: real-life data from the Swedish Heart Failure Registry 2013–2020
Source: Clin Res Cardiol. 2024 Aug 26;113(9):1355–68. doi: 10.1007/s00392-024-02498-z (PMC11371852; doi:10.1007/s00392-024-02498-z)

**Supplement Figure 1. Flow diagram detailing inclusion and exclusion criteria**


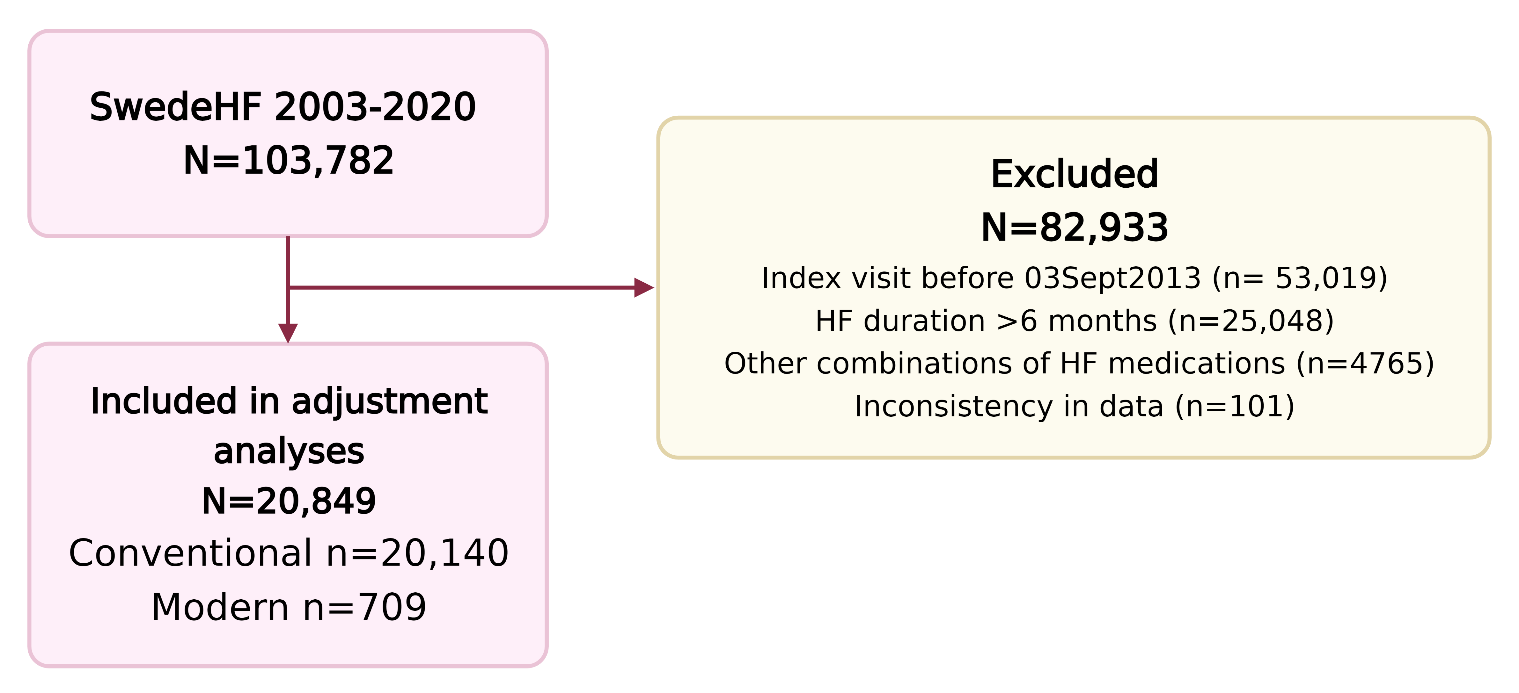

Supplement: Supplementary file 1 — Supplementary file1 (DOCX 98 KB) [file 392_2024_2498_MOESM1_ESM.docx]
