# Supplementary material for: Modern heart failure treatment is superior to conventional treatment across the left ventricular ejection spectrum: real-life data from the Swedish Heart Failure Registry 2013–2020
Source: Clin Res Cardiol. 2024 Aug 26;113(9):1355–68. doi: 10.1007/s00392-024-02498-z (PMC11371852; doi:10.1007/s00392-024-02498-z)

**Supplement Figure** **2. Cumulative incidence curve for conventional vs. modern HF therapy in the overall cohort by LVEF category (A) ischemic etiology (B) age category (C) sex (D)**

**Abbreviations:** CV=cardiovascular, LVEF=left ventricular ejection fraction, HF=heart failure

A.


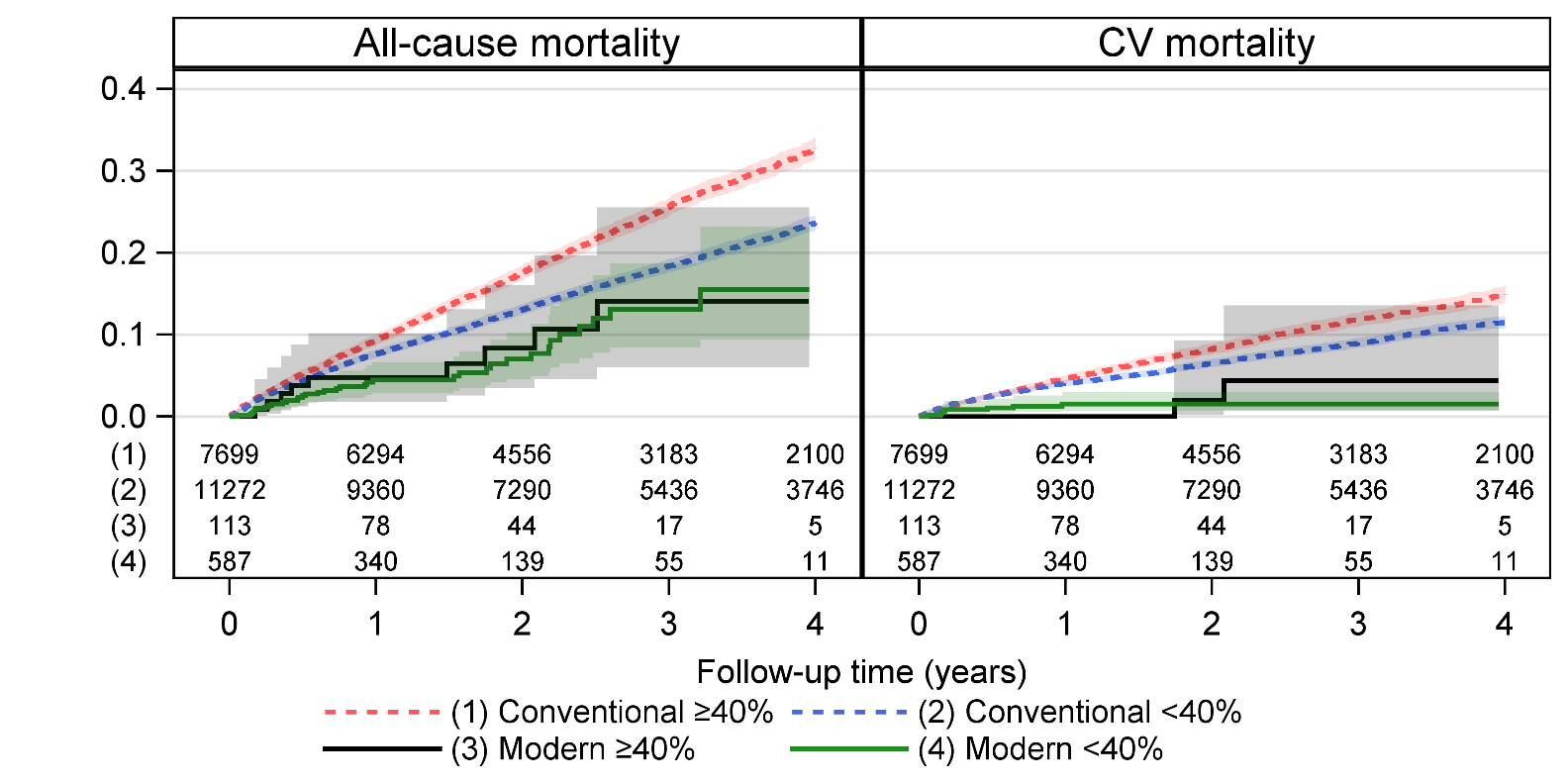


B.


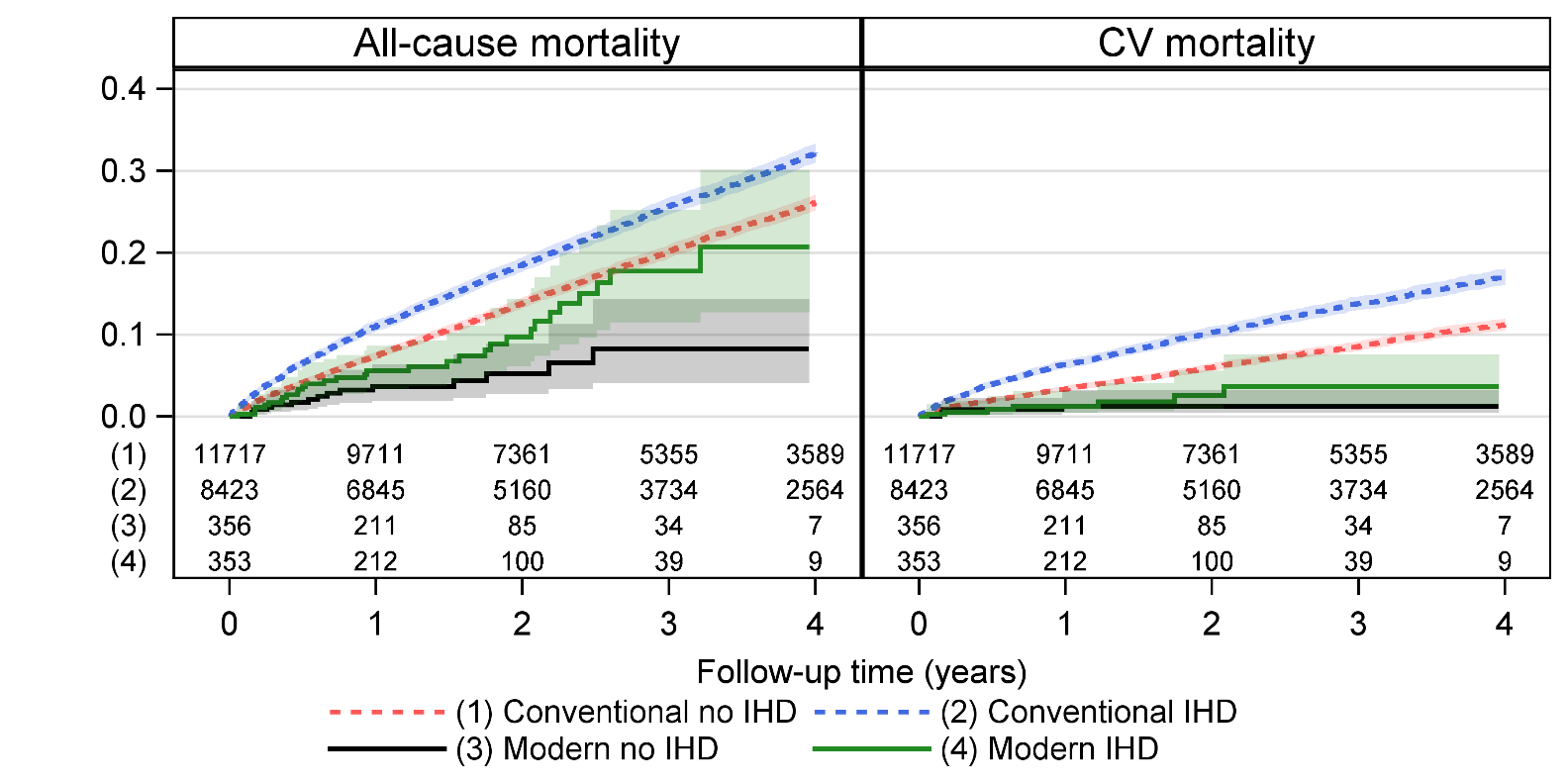


C.


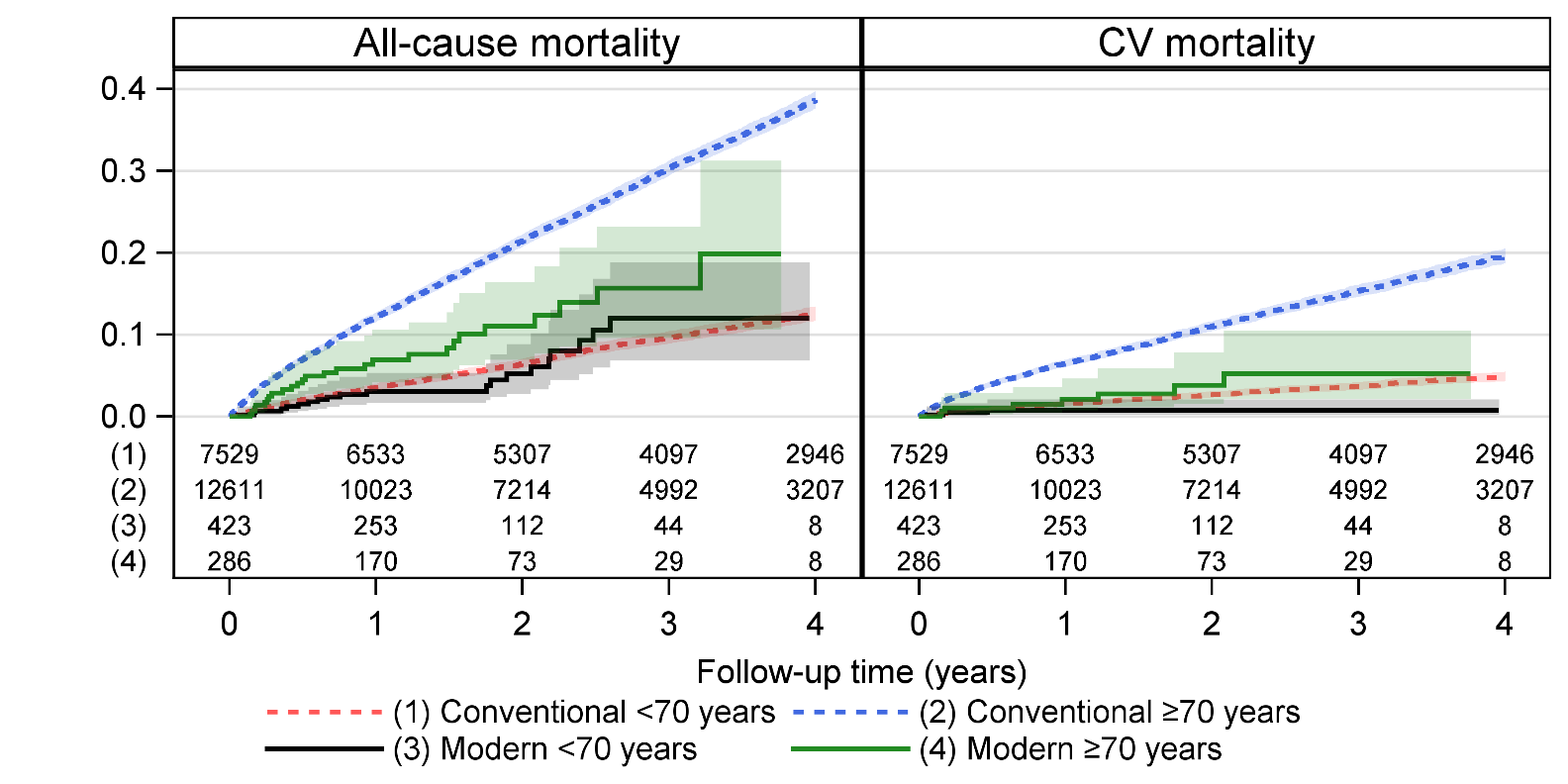
D.
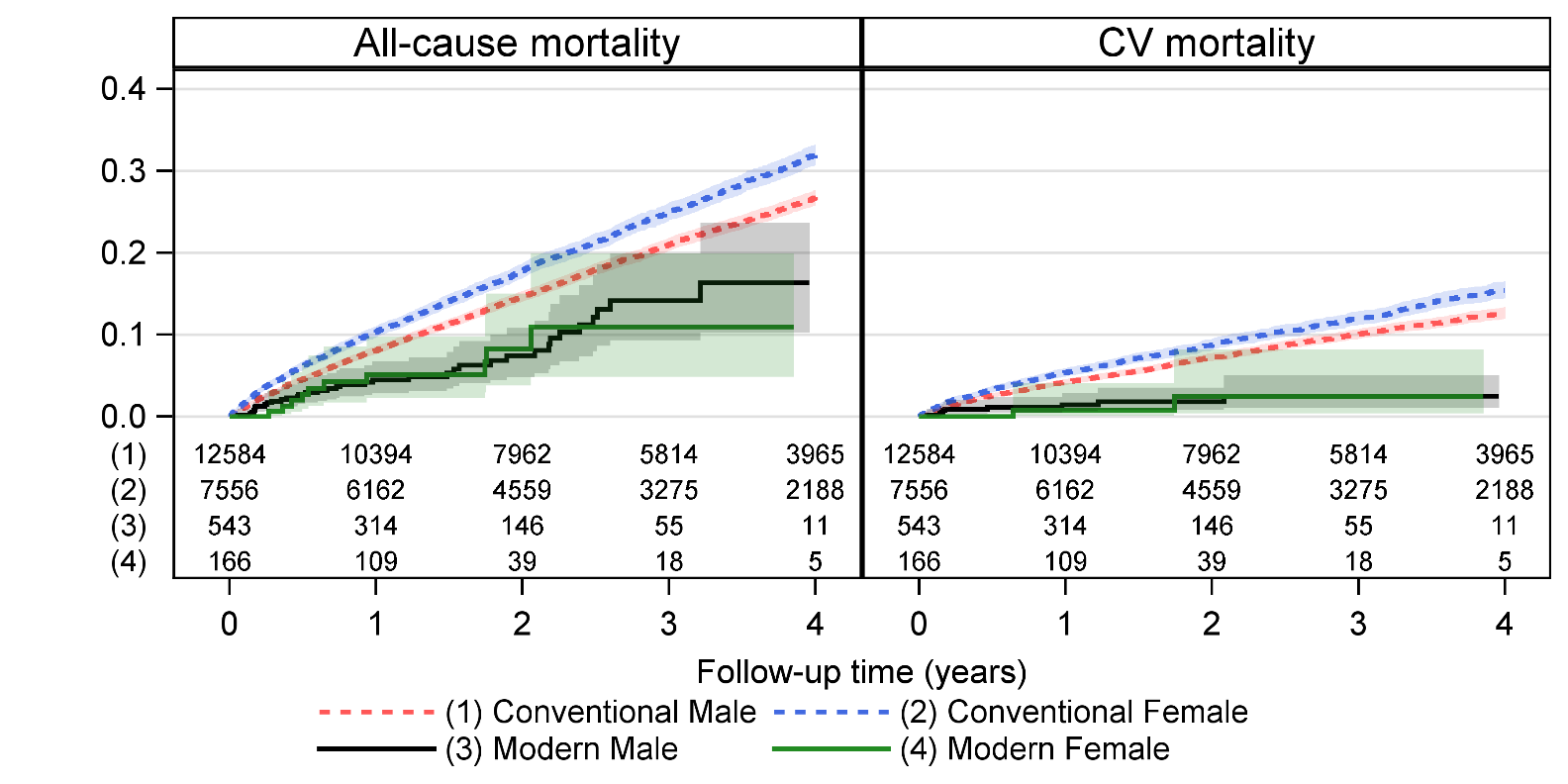

Supplement: Supplementary file 2 — Supplementary file2 (DOCX 411 KB) [file 392_2024_2498_MOESM2_ESM.docx]
