# Supplementary material for: Modern heart failure treatment is superior to conventional treatment across the left ventricular ejection spectrum: real-life data from the Swedish Heart Failure Registry 2013–2020
Source: Clin Res Cardiol. 2024 Aug 26;113(9):1355–68. doi: 10.1007/s00392-024-02498-z (PMC11371852; doi:10.1007/s00392-024-02498-z)
Supplement: Supplementary file 3 — Supplementary file3 (DOCX 15 KB) [file 392_2024_2498_MOESM3_ESM.docx]

**Supplementary Table 1 Comorbidities before or at index visit.**

|  | **National Patient Register ICD-10 codes (in- and outpatient, main and sub-diagnoses)** | **SwedeHF** |
| --- | --- | --- |
| ***Comorbidities (before or at index visit)*** |  |  |
| Hypertension | I10–I13, I15 | Yes |
| Atrial fibrillation | I48 | Yes |
| Chronic obstructive pulmonary disease | J40–J44 | Yes |
| Diabetes mellitus | E10–E14 | Yes |
| Stroke/TIA | I61–I64, G458–G459 | No |
| Psychiatric diagnoses in the past 3 years before admission | F | No |
| Musculoskeletal diseases in the past 3 years before admission | M05–M12, M15–M19, M30–M36, M45–M46, M80–M85 | No |
| Malignant cancer in the past 3 years before admission | C00–C26, C30–C34, C37–C41, C43, C45–C58, C60–C76, C81–C85, C88, C90–C97 | No |
| PCI | FNG | Yes |
| CABG | FNA, FNB, FNC, FND, FNE, FNF, FNH, Z951, Z955 | Yes |
| DCM | I420 | Yes |
| Myocardial infarction | I21, I22 | No |
| Angina | I20 | No |
| Valve surgery | FMD10, FMD00, FMA32, FMD96, FMW96, FMA20, FMC96, FMD30, FMC00 | Yes |
| Valve disease | No | Yes |
| Ischemic heart disease | CABG, PCI, MI, angina | Yes |
|  | **Cause of Death ICD-10 codes (main reason)** | **SwedeHF** |
| ***Outcomes*** |  |  |
| Cardiovascular mortality | I | No |
|  | **Prescribed Drug Register ATC codes (3 months and 6 months after index date)** | **SwedeHF** |
| ***Medications*** |  |  |
| SGLT2i | A10BK | No |
| Beta-blocker | C07AB | Yes |
| ACEi/ARB | C09AA, C09CA | Yes |
| ARNI | C09DX04 | Yes |
| MRA | C03DA | Yes |
| Diuretics | No | Yes |
| ICD = International classification of disease, ATC = Anatomic Therapeutic Chemical, SwedeHF = Swedish Heart Failure Registry, TIA = transient ischemic attack, SGLT2i = sodium-glucose cotransporter 2 inhibitor, ACEi = angiotensin-converting enzyme inhibitor, ARB = angiotensin receptor blocker, ARNI = angiotensin receptor neprilysin inhibitor, MRA = mineralocorticoid receptor antagonist. CABG=coronary artery bypass graft surgery, PCI=percutaneous coronary intervention, DCM=dilated cardiomyopathy | | |
