# Supplementary material for: Modern heart failure treatment is superior to conventional treatment across the left ventricular ejection spectrum: real-life data from the Swedish Heart Failure Registry 2013–2020
Source: Clin Res Cardiol. 2024 Aug 26;113(9):1355–68. doi: 10.1007/s00392-024-02498-z (PMC11371852; doi:10.1007/s00392-024-02498-z)
Supplement: Supplementary file 4 — Supplementary file4 (DOCX 23 KB) [file 392_2024_2498_MOESM4_ESM.docx]

**Supplementary Table 2:** **Patient demographics, clinical data, and comorbidities at the index visit in the SwedeHF comparing modern vs. conventional HF therapy by LVEF (All newly debuted HF patients with index visit from 03Sep2013 onwards).**

|  | **LVEF ≥40%** | | | **LVEF <40%** | | |
| --- | --- | --- | --- | --- | --- | --- |
| **Variable** | **Conventional N=7699** | **Modern N=113** | **P-value** | **Conventional N=11272** | **Modern N=587** | **P-value** |
| ***Patient demographics*** |  |  |  |  |  |  |
| Sex |  |  | 0.0004 |  |  | <.0001 |
| Male | 4292 (55.7%) | 82 (72.6%) |  | 7726 (68.5%) | 454 (77.3%) |  |
| Female | 3407 (44.3%) | 31 (27.4%) |  | 3546 (31.5%) | 133 (22.7%) |  |
| Age at SwedeHF index visit (admission for inpatient care) | 74.2±11.8 76 (18 - 99) n=7699 | 69.1±10.9 71 (31 - 90) n=113 | <.0001 | 70.3±12.2 72 (19 - 99) n=11272 | 64.2±12.2 66 (23 - 91) n=587 | <.0001 |
| Age at admission |  |  | <.0001 |  |  | <.0001 |
| <70 years | 2304 (29.9%) | 54 (47.8%) |  | 5012 (44.5%) | 365 (62.2%) |  |
| $\geq$70 years | 5395 (70.1%) | 59 (52.2%) |  | 6260 (55.5%) | 222 (37.8%) |  |
| ***Clinical data at the index visit*** |  |  |  |  |  |  |
| Weight (kg) | 81.6±19.1 80 (11 - 191) n=7099 | 93.4±20.9 91 (59 - 190) n=102 | <.0001 | 81.7±19.0 80 (10 - 210) n=10661 | 89.3±21.3 86 (39 - 250) n=560 | <.0001 |
| Body mass index (kg/m^2^) | 27.9±5.7 27.0 (4.0 - 83.3) n=6163 | 30.5±5.9 30.2 (19.9 - 51.6) n=93 | <.0001 | 27.1±5.4 26.3 (3.7 - 62.9) n=9233 | 29.0±6.2 28.0 (16.0 - 71.5) n=524 | <.0001 |
| Body mass index (kg/m^2^) |  |  | <.0001 |  |  | <.0001 |
| <18.5 | 112 (1.8%) | 0 (0.0%) |  | 242 (2.6%) | 7 (1.3%) |  |
| 18.5-25 | 1924 (31.2%) | 18 (19.4%) |  | 3356 (36.3%) | 127 (24.2%) |  |
| >25-30 | 2305 (37.4%) | 28 (30.1%) |  | 3354 (36.3%) | 194 (37.0%) |  |
| >30-35 | 1134 (18.4%) | 29 (31.2%) |  | 1531 (16.6%) | 117 (22.3%) |  |
| >35 | 691 (11.2%) | 18 (19.4%) |  | 752 (8.1%) | 79 (15.1%) |  |
| Missing | 1533 | 20 |  | 2037 | 63 |  |
| Systolic blood pressure (mmHg, only for outpatient visits) | 131.8±20.9 130 (10 - 223) n=4979 | 125.8±22.4 125 (12 - 168) n=84 | 0.048 | 126.6±20.8 125 (12 - 240) n=7639 | 119.9±19.1 120 (75 - 198) n=463 | <.0001 |
| Diastolic blood pressure (mmHg, only for outpatient visits) | 75.3±11.6 75 (35 - 127) n=4972 | 73.8±12.0 73 (40 - 111) n=84 | 0.30 | 75.5±12.7 75 (38 - 180) n=7616 | 73.9±11.2 73 (45 - 120) n=463 | 0.013 |
| Heart rate (bpm, only for outpatient visits) | 71.6±14.9 70 (30 - 170) n=4931 | 72.9±14.5 70 (47 - 129) n=85 | 0.40 | 74.2±16.6 72 (36 - 170) n=7601 | 74.8±15.2 74 (42 - 140) n=458 | 0.12 |
| NYHA functional class |  |  | 0.42 |  |  | 0.0005 |
| I | 797 (15.2%) | 12 (13.3%) |  | 1009 (11.8%) | 47 (9.3%) |  |
| II | 2852 (54.4%) | 46 (51.1%) |  | 4646 (54.3%) | 250 (49.2%) |  |
| III | 1535 (29.3%) | 32 (35.6%) |  | 2771 (32.4%) | 202 (39.8%) |  |
| IV | 61 (1.2%) | 0 (0.0%) |  | 124 (1.5%) | 9 (1.8%) |  |
| Missing | 2454 | 23 |  | 2722 | 79 |  |
| LVEF (%) |  |  | 0.07 |  |  | <.0001 |
| 30-<40% | 3108 (40.4%) | 36 (31.9%) |  | 6038 (53.6%) | 233 (39.7%) |  |
| <30% | 4591 (59.6%) | 77 (68.1%) |  | 5234 (46.4%) | 354 (60.3%) |  |
| Potassium (mmol/L, only for outpatient visits) | 4.22±0.41 4.2 (2.2 - 6.1) n=4880 | 4.26±0.42 4.3 (3.3 - 5.2) n=84 | 0.24 | 4.27±0.41 4.3 (2.5 - 6.5) n=7547 | 4.33±0.42 4.3 (3.2 - 5.8) n=457 | 0.0006 |
| NT-proBNP (pg/ml) | 3377.3±5192.7 1870 (1 - 80000) n=5761 | 2471.3±3058.2 1493 (60 - 19793) n=96 | 0.042 | 4980.7±6973.0 2697 (9 - 111601) n=8467 | 3218.4±3883.7 1972 (60 - 32365) n=496 | <.0001 |
| NT-proBNP cat. (pg/ml) |  |  | 0.047 |  |  | <.0001 |
| ≥900 | 1604 (27.8%) | 36 (37.5%) |  | 1581 (18.7%) | 127 (25.6%) |  |
| >900-2500 | 1911 (33.2%) | 32 (33.3%) |  | 2439 (28.8%) | 161 (32.5%) |  |
| >2500-5000 | 1221 (21.2%) | 13 (13.5%) |  | 1958 (23.1%) | 118 (23.8%) |  |
| >5000 | 1025 (17.8%) | 15 (15.6%) |  | 2489 (29.4%) | 90 (18.1%) |  |
| Missing | 1938 | 17 |  | 2805 | 91 |  |
| eGFR (CKD-EPI) | 68.0±21.3 69 (4 - 213) n=4907 | 72.2±21.4 73 (20 - 129) n=83 | 0.09 | 70.3±20.8 71 (5 - 285) n=7572 | 75.1±20.6 77 (22 - 142) n=459 | <.0001 |
| eGFR (CKD-EPI, cat.) |  |  | 0.25 |  |  | 0.0042 |
| <60 | 1741 (35.5%) | 24 (28.9%) |  | 2362 (31.2%) | 114 (24.8%) |  |
| ≥60 | 3166 (64.5%) | 59 (71.1%) |  | 5210 (68.8%) | 345 (75.2%) |  |
| Missing | 2792 | 30 |  | 3700 | 128 |  |
| CKD stages |  |  | 0.052 |  |  | <.0001 |
| CKD stage 1 (eGFR ≥90, Normal and high) | 734 (15.0%) | 18 (21.7%) |  | 1329 (17.6%) | 114 (24.8%) |  |
| CKD stage 2 (eGFR 60-<90, Mild reduction, normal range for young adult) | 2432 (49.6%) | 41 (49.4%) |  | 3881 (51.3%) | 231 (50.3%) |  |
| CKD stage 3 (eGFR 30-<60, Moderate reduction) | 1551 (31.6%) | 23 (27.7%) |  | 2148 (28.4%) | 108 (23.5%) |  |
| CKD stage 4 (eGFR 15-<30, Severe reduction) | 171 (3.5%) | 1 (1.2%) |  | 180 (2.4%) | 6 (1.3%) |  |
| CKD stage 5 (eGFR <15, Kidney failure) | 19 (0.4%) | 0 (0.0%) |  | 34 (0.4%) | 0 (0.0%) |  |
| Missing | 2792 | 30 |  | 3700 | 128 |  |
| ***Medical history at index visit*** |  |  |  |  |  |  |
| IHD | 3424 (44.5%) | 69 (61.1%) | 0.0005 | 4493 (39.9%) | 281 (47.9%) | 0.0001 |
| Valve disease or surgery | 1418 (18.4%) | 15 (13.3%) | 0.18 | 1271 (11.3%) | 38 (6.5%) | 0.0001 |
| Hypertension (NPReg/SwedeHF) | 5844 (75.9%) | 102 (90.3%) | 0.0002 | 6790 (60.2%) | 396 (67.5%) | 0.0004 |
| Atrial fibrillation (NPReg/SwedeHF) | 4348 (56.5%) | 58 (51.3%) | 0.29 | 5334 (47.3%) | 213 (36.3%) | <.0001 |
| Chronic obstructive lunch disease (NPReg/SWEDEHF) | 1278 (16.6%) | 24 (21.2%) | 0.20 | 1645 (14.6%) | 71 (12.1%) | 0.10 |
| Diabetes mellitus (NPReg/SWEDEHF) | 1972 (25.6%) | 88 (77.9%) | <.0001 | 2288 (20.3%) | 324 (55.2%) | <.0001 |
| Blood diseases (NPReg) | 1622 (21.1%) | 31 (27.4%) | 0.10 | 1686 (15.0%) | 87 (14.8%) | 1.00 |
| Stroke/TIA (NPReg) | 1043 (13.5%) | 19 (16.8%) | 0.33 | 1190 (10.6%) | 62 (10.6%) | 1.00 |
| Psychiatric diagnoses past 3 years before admission (NPReg) | 886 (11.5%) | 23 (20.4%) | 0.0070 | 1780 (15.8%) | 94 (16.0%) | 0.86 |
| Musculoskeletal diseases past 3 years before admission (NPReg) | 1567 (20.4%) | 20 (17.7%) | 0.56 | 1680 (14.9%) | 72 (12.3%) | 0.08 |
| Malignant cancer past 3 years before admission (NPReg) | 879 (11.4%) | 7 (6.2%) | 0.10 | 1099 (9.7%) | 52 (8.9%) | 0.52 |
| ICD |  |  | 0.11 |  |  | <.0001 |
| No | 7507 (99.0%) | 110 (97.3%) |  | 10928 (97.5%) | 543 (93.0%) |  |
| Yes | 75 (1.0%) | 3 (2.7%) |  | 284 (2.5%) | 41 (7.0%) |  |
| Missing | 117 | 0 |  | 60 | 3 |  |
| CRT |  |  | 0.011 |  |  | 0.053 |
| No | 7553 (99.6%) | 110 (97.3%) |  | 11048 (98.5%) | 569 (97.4%) |  |
| Yes | 29 (0.4%) | 3 (2.7%) |  | 164 (1.5%) | 15 (2.6%) |  |
| Missing | 117 | 0 |  | 60 | 3 |  |
| ***Medications*** |  |  |  |  |  |  |
| ARNI dispensed 3 m before to 6 m after index visit | 0 (0.0%) | 31 (27.4%) | <.0001 | 0 (0.0%) | 396 (67.5%) | <.0001 |
| ACEi/ARB dispensed 3 m before to 6 m after index visit | 7699 (100.0%) | 87 (77.0%) | <.0001 | 11272 (100.0%) | 280 (47.7%) | <.0001 |
| SGLT2 inhibitors dispensed 3 m before to 6 m after index visit | 0 (0.0%) | 88 (77.9%) | <.0001 | 0 (0.0%) | 322 (54.9%) | <.0001 |
| Diuretics | 4166 (70.5%) | 79 (74.5%) | 0.39 | 5807 (71.4%) | 438 (75.4%) | 0.040 |
| SwedeHF=Swedish heart failure registry, NYHA=New York heart association, LVEF=left ventricular ejection fraction, eGFR=estimated glomerular filtration rate, CKD-EPI=chronic kidney disease epidemiology collaboration, CKD= chronic kidney disease, ICD =implantable cardioverter-defibrillator, CRT=cardiac resynchronization therapy, ARNI=angiotensin receptor neprilysin inhibitor, ACEi=angiotensin-converting enzyme inhibitor, ARB=angiotensin receptor blocker, SGLT2i = sodium-glucose cotransporter 2 inhibitor, NT-proBNP= N-terminal pro B-type natriuretic peptide, IHD=Ischemic Heart Disease, NPReg=National patient register, m=month.  Data are presented as mean±standard deviation, median (range) and number of observations, or number (percentage). For the test between two groups for dichotomous variables, Fisher’s exact test was used, for ordered categorical variables the Mantel-Haenszel chi-square trend test, and for continuous variables the Mann-Whitney U test. | | | | | | |
