# Supplementary material for: Modern heart failure treatment is superior to conventional treatment across the left ventricular ejection spectrum: real-life data from the Swedish Heart Failure Registry 2013–2020
Source: Clin Res Cardiol. 2024 Aug 26;113(9):1355–68. doi: 10.1007/s00392-024-02498-z (PMC11371852; doi:10.1007/s00392-024-02498-z)
Supplement: Supplementary file 5 — Supplementary file5 (DOCX 19 KB) [file 392_2024_2498_MOESM5_ESM.docx]

**Supplementary Table 3: Patient demographics, clinical data and comorbidities at the index visit in the SwedeHF comparing modern vs. conventional HF therapy: 1:1 propensity score-matched cohort including ARNI in the matching procedure.**

| **Variable** | **Conventional N=279** | **Modern N=279** | **P-value** |
| --- | --- | --- | --- |
| ***Patient demographics*** |  |  |  |
| Sex |  |  | 0.49 |
| Male | 205 (73.5%) | 213 (76.3%) |  |
| Female | 74 (26.5%) | 66 (23.7%) |  |
| Age at SwedeHF index visit (admission for inpatient care) | 67.1±12.3 69 (24 - 90) n=279 | 67.6±10.7 69 (25 - 90) n=279 | 0.95 |
| Age at admission |  |  | 1.00 |
| <70 years | 148 (53.0%) | 147 (52.7%) |  |
| ≥70 years | 131 (47.0%) | 132 (47.3%) |  |
| ***Clinical data at index visit*** |  |  |  |
| Weight (kg) | 91.3±20.2 89 (53 - 170) n=259 | 92.8±21.9 90 (46 - 250) n=257 | 0.37 |
| Body mass index (kg/m^2^) | 30.2±5.6 28.9 (18.4 - 51.3) n=242 | 30.3±6.3 29.6 (16.2 - 71.5) n=243 | 0.89 |
| Body mass index (kg/m^2^) |  |  | 0.98 |
| <18.5 | 1 (0.4%) | 2 (0.8%) |  |
| 18.5-25 | 39 (16.1%) | 42 (17.3%) |  |
| >25-30 | 96 (39.7%) | 85 (35.0%) |  |
| >30-35 | 57 (23.6%) | 68 (28.0%) |  |
| >35 | 49 (20.2%) | 46 (18.9%) |  |
| Missing | 37 | 36 |  |
| Systolic blood pressure (mmHg, only for outpatient visits) | 127.4±19.8 127 (80 - 170) n=176 | 125.2±19.4 125 (71 - 193) n=188 | 0.16 |
| Diastolic blood pressure (mmHg, only for outpatient visits) | 74.5±11.4 75 (49 - 115) n=176 | 74.2±10.4 75 (45 - 111) n=188 | 0.76 |
| Heart rate (bpm, only for outpatient visits) | 76.5±17.2 74 (43 - 150) n=180 | 76.4±15.0 76 (49 - 130) n=189 | 0.62 |
| NYHA functional class |  |  | 0.80 |
| I | 14 (6.5%) | 16 (7.6%) |  |
| II | 131 (60.9%) | 121 (57.3%) |  |
| III | 69 (32.1%) | 73 (34.6%) |  |
| IV | 1 (0.5%) | 1 (0.5%) |  |
| Missing | 64 | 68 |  |
| LVEF (%) |  |  | 0.42 |
| ≥50% | 40 (14.4%) | 31 (11.4%) |  |
| 40-<50% | 52 (18.7%) | 51 (18.7%) |  |
| 30-<40% | 91 (32.7%) | 95 (34.8%) |  |
| <30% | 95 (34.2%) | 96 (35.2%) |  |
| Missing | 1 | 6 |  |
| Potassium (mmol/L, only for outpatient visits) | 4.25±0.42 4.2 (3.3 - 5.8) n=176 | 4.31±0.43 4.3 (3.3 - 5.8) n=182 | 0.14 |
| NT-proBNP (pg/ml) | 3394.3±5443.3 1777 (42 - 51911) n=235 | 3026.5±3420.2 1959 (60 - 22000) n=221 | 0.64 |
| NT-proBNP cat. (pg/ml) |  |  | 0.46 |
| ≥900 | 67 (28.5%) | 53 (24.0%) |  |
| >900-2500 | 78 (33.2%) | 79 (35.7%) |  |
| >2500-5000 | 52 (22.1%) | 52 (23.5%) |  |
| >5000 | 38 (16.2%) | 37 (16.7%) |  |
| Missing | 44 | 58 |  |
| eGFR (CKD-EPI) | 74.8±22.0 75 (7 - 138) n=176 | 76.0±21.2 80 (20 - 142) n=181 | 0.83 |
| eGFR (CKD-EPI) |  |  | 0.80 |
| <60 | 39 (22.2%) | 43 (23.8%) |  |
| ≥60 | 137 (77.8%) | 138 (76.2%) |  |
| Missing | 103 | 98 |  |
| CKD stages |  |  | 0.73 |
| CKD stage 1 (eGFR ≥90, Normal and high) | 45 (25.6%) | 47 (26.0%) |  |
| CKD stage 2 (eGFR 60-<90, Mild reduction, normal range for young adult) | 92 (52.3%) | 91 (50.3%) |  |
| CKD stage 3 (eGFR 30-<60, Moderate reduction) | 33 (18.8%) | 42 (23.2%) |  |
| CKD stage 4 (eGFR 15-<30, Severe reduction) | 4 (2.3%) | 1 (0.6%) |  |
| CKD stage 5 (eGFR <15, Kidney failure) | 2 (1.1%) | 0 (0.0%) |  |
| Missing | 103 | 98 |  |
| ***Medical history at index visit*** |  |  |  |
| IHD | 157 (56.3%) | 172 (61.6%) | 0.23 |
| Valve disease or surgery | 19 (6.8%) | 18 (6.5%) | 1.00 |
| Hypertension (NPReg/SwedeHF) | 230 (82.4%) | 233 (83.5%) | 0.82 |
| Atrial fibrillation (NPReg/SwedeHF) | 116 (41.6%) | 115 (41.2%) | 1.00 |
| Chronic obstructive lunch disease (NPReg/SWEDEHF) | 51 (18.3%) | 45 (16.1%) | 0.58 |
| Diabetes mellitus (NPReg/SwedeHF) | 262 (93.9%) | 262 (93.9%) | 1.00 |
| Blood diseases (NPReg) | 63 (22.6%) | 56 (20.1%) | 0.54 |
| Stroke/TIA (NPReg) | 38 (13.6%) | 32 (11.5%) | 0.52 |
| Psychiatric diagnoses past 3 years before admission (NPReg) | 47 (16.8%) | 42 (15.1%) | 0.64 |
| Musculoskeletal diseases past 3 years before admission (NPReg) | 46 (16.5%) | 35 (12.5%) | 0.23 |
| Malignant cancer past 3 years before admission (NPReg) | 36 (12.9%) | 21 (7.5%) | 0.050 |
| ICD |  |  | 0.82 |
| No | 267 (96.0%) | 269 (96.8%) |  |
| Yes | 11 (4.0%) | 9 (3.2%) |  |
| Missing | 1 | 1 |  |
| CRT |  |  | 1.00 |
| No | 276 (99.3%) | 275 (98.9%) |  |
| Yes | 2 (0.7%) | 3 (1.1%) |  |
| Missing | 1 | 1 |  |
| ***Medications*** |  |  |  |
| ARNI dispensed 3 m before to 6 m after index visit | 0 (0.0%) | 0 (0.0%) |  |
| ACEi/ARB dispensed 3 m before to 6 m after index visit | 279 (100.0%) | 279 (100.0%) |  |
| SGLT2 inhibitors dispensed 3 m before to 6 m after index visit | 0 (0.0%) | 279 (100.0%) |  |
| Diuretics | 162 (77.5%) | 197 (73.2%) | 0.29 |
| AF=Atrial fibrillation, BMI=Body mass index, SwedeHF=Swedish heart failure registry, NYHA=New York heart association, LVEF=left ventricular ejection fraction, eGFR=estimated glomerular filtration rate, CKD= chronic kidney disease, CKD-EPI = chronic kidney disease epidemiology collaboration, ICD =implantable cardioverter-defibrillator, CRT=cardiac resynchronization therapy, ARNI=angiotensin receptor neprilysin inhibitor, ACEi=angiotensin-converting enzyme inhibitor, ARB=angiotensin receptor blocker, SGLT2 = sodium-glucose cotransporter 2, NT-proBNP= N-terminal pro-B-type natriuretic peptide, IHD=ischemic heart disease, NPReg=National patient register, m=month.  Data are presented as mean±standard deviation, median (range), and number of observations or number (percentage). For test between two groups for dichotomous variables, Fisher's exact test was used; for ordered categorical variables, the Mantel-Haenszel chi-square trend test was applied, and for continuous variables, the Mann-Whitney U test was executed.  Propensity score matching is performed including age, sex, BMI, NYHA, LVEF, NT-proBNP, eGFR, IHD, valve surgery, hypertension, AF, diabetes, ICD, CRT, and ARNI. | | | |
